# The efficacy and mechanisms of low-intensity transcranial ultrasound stimulation on pain: a systematic review of human and animal studies

**DOI:** 10.1186/s10194-025-02096-y

**Published:** 2025-07-22

**Authors:** Hao-Ran Xu, Yiu-Liang Yi, Chuan Xue, Zi-Qi Guo, Li Ding, Jie Jia

**Affiliations:** 1 Department of Rehabilitation Medicine, Huashan Hospital, Fudan University, Shanghai, China; 2 National Clinical Research Center for Aging and Medicine, Huashan Hospital, Fudan University, Shanghai, China

**Keywords:** Transcranial ultrasound, Low-intensity transcranial ultrasound, Pain, Neuromodulation, Effectiveness

## Abstract

**Background:**

Low-intensity transcranial ultrasound stimulation (LITUS) is an emerging non-invasive neuromodulation technique for pain treatment, with the unique ability to modulate deep brain nuclei associated with pain. The aim of this study is to systematically review and summarize the evidence for the efficacy of LITUS in pain management and to elucidate the potential mechanisms underlying its analgesic effects.

**Methods:**

A systematic search was conducted across five databases up to Mar 31st, 2025. Controlled studies in both human and animal subjects were included. Two independent reviewers completed the screening and risk of bias assessment process following predefined inclusion and exclusion criteria.

**Results:**

A total of thirteen studies were included in the review. These studies demonstrated LITUS’s potential in managing various types of pain among different populations and animal models, particularly targeting the anterior cingulate cortex, thalamus, insular cortex, primary sensorimotor cortex, and periaqueductal gray. Most included studies showed positive effects and verified the safety of LITUS on pain, reporting few adverse effects.

**Conclusions:**

LITUS is an effective and non-invasive tool for pain regulation in animals and humans, enabling precise modulation of deep brain circuits. Analgesic effects may be affected by pain-related risk factors, insufficient dosage, suboptimal protocols, and target selection. Initial evidence has highlighted the direct link between LITUS parameters, brain region responses, and pain behavior. Modulation of brain excitatory, nociceptive circuit, electrophysiological response, autonomic response, biochemistry, neuroinflammation, and psychology are proposed as the potential mechanisms underlying the efficacy of LITUS. More high-quality research is urgently needed to advance clinical LITUS use and reveal its mechanisms.

**Supplementary Information:**

The online version contains supplementary material available at 10.1186/s10194-025-02096-y.

## Highlights

• LITUS shows promising applications in pain management based on existing research.

• The characteristics of LITUS enable precise modulation of deep pain-related nuclei.

• Current research primarily originates from animal experiments and provoked pain in healthy subjects.

• Some studies have begun to explore the mechanisms of LITUS for pain relief, but much remains unclear.

• High-quality studies are needed to verify the analgesic effect LITUS and optimize stimulation protocols.

**Supplementary Information:**

The online version contains supplementary material available at 10.1186/s10194-025-02096-y.

## Introduction

Pain is a complex sensory and emotional experience associated with actual or potential tissue damage, and it is a common symptom that prompts individuals to seek medical attention [1]. Globally, 20% of adults are diagnosed with pain, and 10% suffer from chronic pain (CP), with some countries reporting rates as high as 20–25% [2]. In the US, pain inflicts an economic loss of $560 to $635 billion annually, highlighting its importance as a public health issue [3, 4]. Currently, the mainstream approach to manage pain involves pharmacological interventions, such as opioids and nonsteroidal anti-inflammatory drugs [5]. However, the substantial side effects, including addiction, limit their application and patient compliance. Moreover, over 60% of patients report that common medications fail to effectively alleviate their pain [6]. Non-invasive brain stimulation (NIBS), including transcranial electrical stimulation (tES) and transcranial magnetic stimulation (TMS), has become an effective approach for regulating brain activity and treating various neurological and mental disorders, including pain. To date, a recent guideline has given an A-level recommendation for the use of TMS, specifically targeting the primary motor cortex (M1) opposite the site of pain, in the treatment of neuropathic pain (NP). This endorsement underscores the recognized role of NIBS in effective pain management [7]. However, pain management in clinical practice often fails to meet satisfaction [8, 9]. One possible reason for the suboptimal outcomes is that pain is a complicated process involving various brain areas and neural circuits [10]. Given the intricate nature of these circuits, modulating the M1 target alone may not be the most effective strategy from a comprehensive, whole-brain perspective. On the other hand, the low spatial resolution and penetration depth of TMS and tES technologies limit the ability to modulate deep brain nuclei involved in pain processing [11]. Deep brain stimulation technique, while overcoming the above challenges, is still limited in clinical application due to its invasive nature.

Transcranial ultrasound stimulation (TUS), especially low-intensity transcranial ultrasound stimulation (LITUS), is a novel neuromodulation technique that has emerged in recent years, which utilizes mechanical waves to stimulate the brain non-invasively [12]. Unlike TUS in high-intensity, which primarily relies on thermal effects for tissue ablation, TUS in low-intensity primarily focuses on neuromodulation through its non-thermal and mechanical effects [13]. Based on the focusing characteristics of ultrasound waves, LITUS can be categorized into focused transcranial ultrasound (tFUS) and unfocused TUS [14]. Typically, tFUS directs ultrasound energy to a specific focal area with single-element spherical transducers or annular arrays. This enables precise modulation of neural activity in both cortical and subcortical areas, with the advantages of high spatial resolution and deep penetration. On the other hand, unfocused TUS deliver ultrasound waves in a more dispersed manner, which may be suitable for studies where broad neural modulation is desired. By adjusting parameters such as pulse repetition frequency (PRF) and duty cycle (DC), LITUS can exert either excitatory or inhibitory effects on neural tissue, making it a versatile tool for treating various disorders [15, 16]. Existing studies have demonstrated its efficacy in various neurological disease, including Parkinson disease [17, 18] and depression [19, 20]. Notably, Qin et al. recently conducted a comprehensive systematic review on LITUS, examining its neuromodulation effects on neurophysiological, neuroimaging, histological, and biochemical outcomes, as well as safety concerns, which has advanced the clinical application of LITUS. This systematic review applied rigorous methodology to underscore the safety and clinical potential of LITUS in various pathological circumstances [11]. However, it must be acknowledged that, given the broad scope of Qin et al.‘s review across multiple outcome measures, the discussion of LITUS’s application in pain management remains preliminary. Moreover, the mechanisms through which LITUS exerts its analgesic effects have yet to be explored. Given the intimate relationship between pain and brain function, the application of LITUS in pain modulation shows promise [21]. Hameroff and colleagues were pioneers in applying unfocused LITUS to CP patients, specifically targeting the frontal-temporal cortex [22]. The results of this landmark experiment showed a trend toward pain reduction, without statistically significance. This may have been partly due to the inability of unfocused LITUS to achieve deep and precise modulation. Recent advances in tFUS have enabled precise modulation of deep brain regions and there’s been a marked growth in animal and human studies employing LITUS to modulate deep pain-related nuclei. Given these advancements, a systematic review is urgently needed to focus on LITUS’s impact on pain through comprehensive searches, explore its analgesic mechanisms, identify influential factors for efficacy, and evaluate safety.

The primary aim of this study is to systematically review the efficacy of LITUS intervention in pain based on existing evidence from both animal and human studies. The second aim is to comprehensively summarize the potential mechanisms of LITUS analgesic effects from multiple perspectives. This review will provide insights into parameters, targets, protocols, and theoretical aspects for the future application of LITUS in pain modulation.

## Methods

This study was conducted in accordance with the Preferred Reporting Items for Systematic Reviews and Meta-Analysis (PRISMA) guidelines for systematic reviews and meta-analyses [23], and the PROSPERO registration number was CRD42024595820.

### Search strategy

To retrieve relevant articles, we carried out extensive searches in five databases: MEDLINE, EMBASE, CENTRAL, CINAHL and WOS, spanning from their inception to March 31 st, 2025, and limited to English-language work. To ensure comprehensive coverage of LITUS-related studies, the keywords combined various TUS-related terms drawn from previous high-quality literature about LITUS [11]. Meanwhile, as this study focuses on the analgesic effects of LITUS, the broad term “pain” was used to include all pain-related studies. For the detailed search strategy and results, please refer to Supplement 1.

### Study selection and data extraction

Titles and abstracts of potential studies were independently assessed by two reviewers (HR-X and YY-L), who then examined the full texts against the predefined inclusion and exclusion criteria. Eligibility criteria for the studies included in this research were as follows: 1) human studies in randomized controlled and crossover design, or animal studies in controlled design [11, 24]; (2) used LITUS targeting any area of the brain to modulate pain; (3) had outcomes of interests including any type of pain. Studies were excluded if they used medium-intensity TUS or high-intensity TUS. Disagreements were resolved through consensus with a third reviewer (JJ). Data extraction was conducted by HR-X and YY-L using two predesigned forms, one for human studies and one for animal studies. The forms captured study information (publication year and authors), type of subjects (participants population for animal studies or models for animal studies), grouping and sample size, LITUS characteristics (target area, stimulation sessions, and parameters), main outcomes (pain-related outcome measures, timepoints, and findings comparing experimental and control groups), and adverse events (AEs) (methods of AEs assessment and outcomes).

### Risk of bias evaluation

The risk of bias (RoB) in each study was independently evaluated by two reviewers (HR-X and YY-L). The RoB in human studies was assessed using the Cochrane Risk of Bias 2 tool (Cochrane RoB 2.0) [25], while the Systematic Review Center for Laboratory Animal Experimentation Risk of Bias (SYRCLE’s RoB) tool was employed for animal studies, respectively [24]. Any disagreements in the bias assessment were resolved by consultation with a third reviewer (JJ).

### Strategy for qualitative synthesis

Due to significant variability among studies in terms of pain-related outcome measures, participant demographics, target areas for treatment, and sonication parameters, conducting a meta-analysis was not feasible [26]. Thus, studies targeting the same brain regions were categorized, and a comparative summary of results between groups was provided. The efficacy of LITUS on pain were divided into four sections based on the targeted brain areas within the studies: anterior cingulate cortex (ACC), insular cortex (IC), thalamus (THAL), and other brain regions. Furthermore, a summary of AEs related to LITUS reported in all included studies was also concluded.

## Results

### Study selection and characteristics

**Fig. 1:**
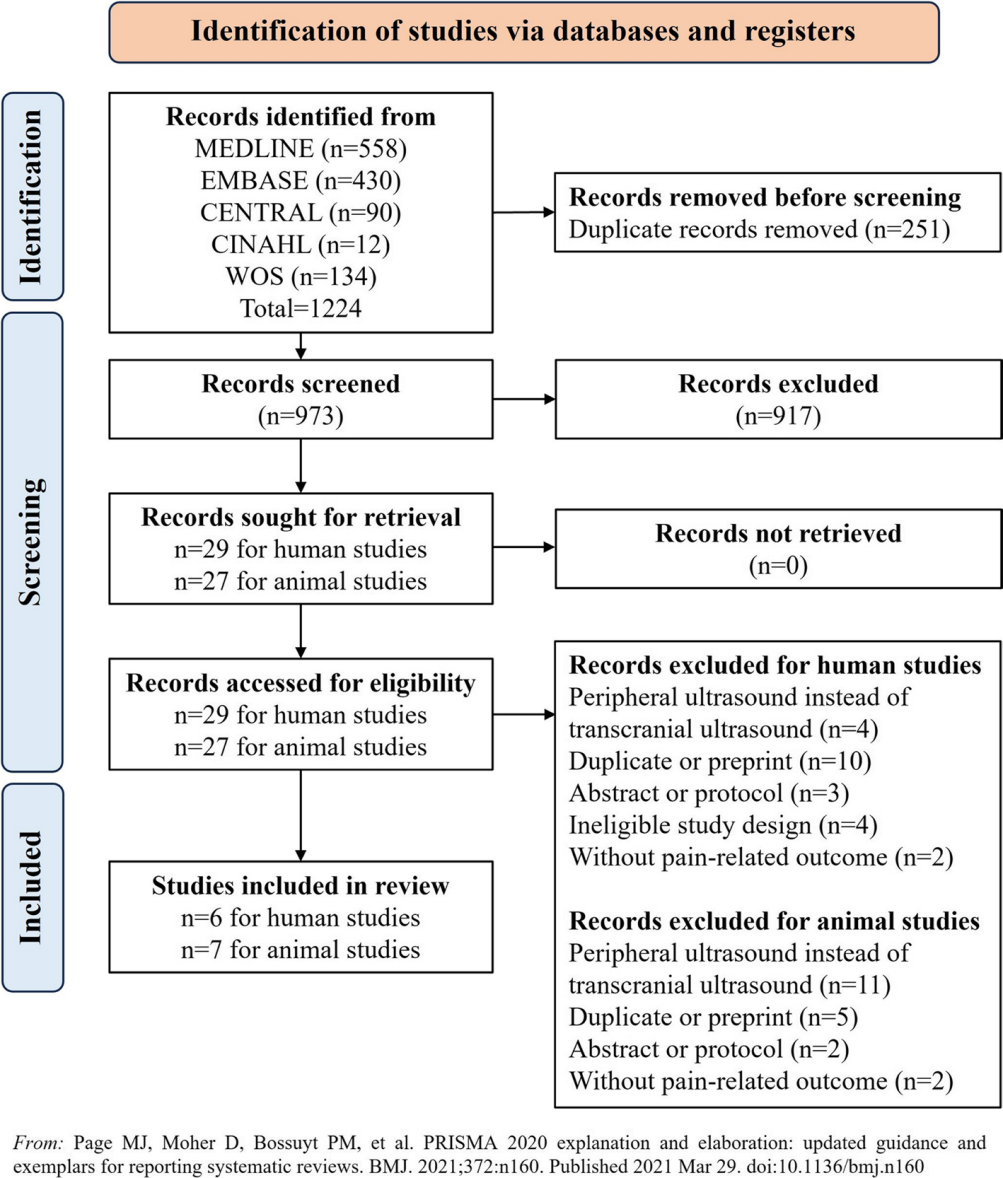
PRISMA framework for study selection process

Following an initial search, a total of 1,224 records were identified, from which 56 studies were selected for full-text screening. Upon thorough examination, 13 studies (including 6 studies based on human subjects and 7 studies on based animal subjects) were eligible for inclusion. The process of study screening is graphically represented in Fig. 1. The reasons for the studies that were excluded during the full-text screening process were provided in Supplement 2. The detailed information of the human and animal studies investigating the analgesic effect of LITUS were demonstrated in Tables 1 and 2, respectively. Of the six human studies included, four involved assessments of healthy subjects through provoked pain [27–30], while the remaining two recruited patients with CP [22, 31]. Multiple pain models were utilized across seven animal studies. The chronic constriction injury (CCI) model, a widely used method to simulate NP via partial ligation of peripheral nerves, was used in two studies [32, 33]. The sickle cell disease (SCD) model, a genetic model expressing human sickle hemoglobin exhibiting CP symptoms, was used in two studies [34, 35]. The remaining studies employed the formalin injection model, which involves injecting formalin solution into a rat’s hindpaw to study inflammatory pain [36] and the adult macaque model for provoked pain [37, 38].

**Table 1:** Characteristics of included human studies

Study	Type of subjects	Grouping (sample size)	Characteristics of LITUS			Main outcomes			Reported adverse event
			Target	Stimulation sessions	Parameters	Pain-related outcome measures	Timepoint of outcome measures	Main results (experimental group compared to the control group)	
Hameroff et al. 2013 [22]	Chronic pain patients	Active group; Sham group (*n* = 31 in the crossover design)	Frontal-temporal cortex (contralateral to pain)	Two sessions of 15-second treatment, one with active treatment and one with sham treatment, in a crossover manner	Unfocused ultrasound: F_0_ = 8 MHz SD = 15s MI = 0.7 ISPPA = 132.43 W/cm^2^ ISPTA = 152 mW/cm^2^	NRS	A total of 4 timepoints: 10 min before and after the first treatment; 40 min after the first treatment; 10 min after the second treatment	Offline effect: NRS↓ at 40 min after the first treatment, indicating a trend towards significance. (*p* = 0.07)	No serious AEs reported during or after the treatment. One subject experienced an exacerbation of a headache following treatment, which quickly subsided without sequelae.
Bardran et al. 2020 [27]	Healthy adults	Active group; Sham group (*n* = 19 in the crossover design)	Right anterior THAL	Two visits at least one week apart: one with active tFUS to the THAL and one with sham stimulation. Each visit contains two 10-minute sessions within the MRI scanner (either active or sham) with a 10-minute interval between sessions	tFUS with inhibitory effect: F_0_ = 650 kHz PRF = 10 Hz PRP = 0.1s Np = 300 pulses PD = 5ms DC = 5% SD = 30s ISI = 30s ISPPA = 14.38 W/cm^2^ ISPTA = 719mW/cm^2^ MI = 0.81	QST, including thermal sensory threshold, thermal pain threshold and thermal pain tolerance	At baseline and after tFUS	Offline effect: Thermal pain thresholds↑	No AEs were observed throughout the experiment.
				10 out of 19 blocks concurrently with pain induction within the second 10-minute session		NRS to CHEP stimuli (7/10 pain intensity at baseline)	Immediately after each pain stimulation	Online effect: No statistical significance	
Strohman et al. 2024 [28]	Healthy human volunteers	tFUS group; Sham group (*n* = 16 in the crossover design)	dACC (MNI: 0, 18, 30)	Two sessions on separate days: one with 40 time-locked CHEPs to the dACC, each lasting for 1 s, and one with sham stimulation	tFUS with inhibitory effect: F_0_ = 500 kHz PRF = 1 kHz DC = 36% Total stimulation duration = 1s ISPPA = 4.34 W/cm^2^ ISPTA = 1.56 W/cm^2^	NRS to CHEP stimulus on dorsum of right hand (5/9 pain intensity at baseline) and the CHEP amplitude to heat stimulus	During the entire stimulation process (40 trials)	Online effect: NRS↓ P2 amplitude↓ SDNN↑ LF/HF ratio↑	No moderate or serious AEs were reported for either condition according to ROS. Minor AEs included unusual feelings, tingling, itching, sleepiness, inattentiveness, twitching, anxious feelings, balance issues, headache, scalp sensations, neck pain, tooth pain, and nausea.
Riis et al. 2024 [31]	Chronic pain patients	Active group; Sham group (*n* = 20 in the crossover design)	ACC	16 brief 30-second tFUS sessions	tFUS with inhibitory effect: F_0_ = 650 kHz PD = 5ms DC = 50% PRF = 1.42 Hz ISPPA = 31.0 W/cm^2^ ISPTA = 0.66 W/cm^2^ MI = 1.2 TI = 0.64	Verbally reported NRS	Immediately after each stimulation	Online effect: Average NRS pain scores↓	No AEs were detected. Side effects were generally mild and resolved within 24 h.
	Chronic pain patients	Active group; Sham group (*n* = 20 in the crossover design)	ACC, specifically 4 personalized targets within the sACC and aMCC for each patient	Two 40-mins sessions on separate days one week apart: one with tFUS to the ACC (twelve 3-minutes sonication) and one with sham stimulation	aMCC: faciliatory sACC: inhibitory F_0_ = 650 kHz PD = 5ms DC = 50% PRF = 1.42 Hz ISPPA = 31.0 W/cm^2^ ISPTA = 0.66 W/cm^2^ MI = 1.2 TI = 0.64	BPI average 24-hour NRS pain intensity scores PROMIS pain intensity scores	From day 1 to day 7 after each stimulation session	Offline effect: NRS↓ from day 1 to day 7 post-intervention Numbers of patients reported a clinically meaningful NRS↓ (Active vs. sham: 75% vs. 15%) Numbers of patients reported NRS↓ greater than 50% (60% vs. 10%) Day 1 after stimulation: Numbers of patients reported a clinically meaningful NRS↓ (60% vs. 15%) Numbers of patients reported NRS↓ greater than 50% (55% vs. 10%) Day 7 after stimulation: Numbers of patients reported a clinically meaningful NRS↓ (60% vs. 20%) Numbers of patients reported NRS↓ greater than 50% (30% vs. 10%) PROMIS pain intensity↓ Numbers of patients reported a clinically meaningful PROMIS pain intensity↓ (55% vs. 17%)	No AEs were detected. Side effects were generally mild and resolved within 24 h.
In et al. 2024 [29]	Healthy adults	AI group; PI group; dACC group; Sham group (*n* = 16 in the crossover design)	Left AI; PI; dACC (0,18,30)	Four 10-minute tFUS were applied to participants in four separate visits, with a minimum of two days between visits.	tFUS with inhibitory effect: F_0_ = 500 kHz PRF = 1 kHz PD = 0.36ms DC = 36% SD = 1s ISI = 5s **For AI and PI target**: External pressure = 380 kPa ISPPA = 4.2 W/cm^2^ ISPTA = 1.5 W/cm^2^ MI = 0.2 **For dACC target**: External pressure = 400 kPa ISPPA = 4.5 W/cm^2^ ISPTA = 1.62 W/cm^2^ MI = 0.23	CPM and TSP task	Before and after the tFUS intervention	Offline effect: tFUS to the PI significantly↓ pain ratings during the TSP protocol tFUS to PI elicited a minor reduction of pain during CPM, but did not reach statistical significance	No serious AEs were reported for any of the interventions, evidenced by ROS. The most reported symptoms were sleepiness and anxiousness.
Legon et al. 2024 [30]	Healthy adults	AI group; PI group; Sham group (*n* = 23 in the crossover design)	Left AI; PI	Participants underwent three separate tFUS sessions, each time-locked to CHEP stimuli for a total of 40 s (1s × 40). One session targeted the AI, another the PI, and a third was a sham treatment, with at least two days between each session.	tFUS with inhibitory effect: F_0_ = 500 kHz PRF = 1 kHz ISPPA = 3.5 W/cm^2^ DC = 36% PD = 0.36ms	Pain ratings to CHEP stimuli N1/P1 peak-to-peak amplitude of the CHEP N1/P1 amplitudes and latencies of the CHEP Time/frequency analysis	Continuously during the testing session, with the pain rating being assessed after each CHEP stimulus	Online effect: tFUS to PI: Pain ratings↓. tFUS to both AI and PI: CHEP peak-to-peak amplitudes↓ tFUS to AI: pain-related power↓ in delta, theta and alpha frequency bands during pain task tFUS to AI: autonomic response SDNN↑ LF power↑	No severe AEs or symptoms were reported for any condition (AI, PI, and Sham) either before or after the intervention. The most frequently reported symptom was sleepiness

*tFUS* Transcranial focused ultrasound stimulation, *F*_0_ Fundamental frequency, *ISPTA* Spatial peak temporal average intensity, *ISPPA* Spatial peak pulse average intensity, *SD* Sonication duration, *PRF* Pulse repetition frequency, *PD* Pulse duration, *DC* Duty cycle, *MI* Mechanical index, *TI* Thermal index, *dACC* Dorsal anterior cingulate cortex, *MNI* Montreal Neurological Institute, *NRS* Numerical rating scale, *CHEP* Contact heat-evoked potential, *AEs* Adverse events, *ROS* Report of symptoms questionnaire, *ACC* Anterior cingulate cortex, *aMCC* Anterior midcingulate cortex, *NRS* Numerical rating scale, *BPI* Brief pain inventory, *PROMIS* Patient-Reported Outcomes Measurement Information System, *THAL* Thalamus, *QST* Quantitative sensory testing, *AI* Anterior insula, *PI* Posterior insula, *CPM* Conditioned pain modulation, *TSP* Temporal summation of pain, *sACC* Subgenual ACC, *SDNN* Standard Deviation of the Normalized NN Intervals, *LF* Low frequency

**Table 2:** Characteristics of included animal studies

Study	Type of subjects	Grouping (sample size)	Characteristics of LITUS			Main outcomes			Reported adverse event
			Target	Stimulation sessions	Parameters	Pain-related outcome measures	Timepoint of outcome measures	Main results (experimental group compared to the control group)	
Feng et al. 2021 [32]	Mouse models of chronic neuropathic pain (CCI models)	tFUS1 group (*n*=6); tFUS2 group (*n*=6); Sham group (*n*=6)	ACC	15 min/ session; 1 session per day for 21 days	tFUS with inhibitory effect (tFUS1): F_0_=3.7 MHz ISPTA=1.598W/cm^2^ ISPPA=15.98W/cm SD=0.4 s ISI=2.6 s PRF=1.5 kHz PD=0.07 ms DC=10%	Mechanical allodynia test; Thermal allodynia test	During the treatment period and 4, 11, 18 days after treatment	tFUS1 group: MWT on the contralateral side↑ 11 days after treatment; tFUS2 group: MWT on the surgical side↑ during follow up; tFUS2 group: MWT and TWT on the contralateral side↑ 4 days after treatment	No abnormal findings in ACC after tFUS, evidenced by histology and temperature evaluation
		tFUS2 group (*n* =12); Sham group (*n*=12)	ACC		tFUS with inhibitory effect (tFUS2): F_0_=3.7 MHz ISPTA=3.498W/cm^2^ ISPPA=34.982W/cm^2^ SD=15 min PRF=1.5 kHz PD=0.07 ms DC=10%	Mechanical allodynia test; Thermal allodynia test	During the treatment period and 3 days after treatment	MWT on the surgical side↑ during treatment period	
Wang et al. 2022 [33]	Chronic neuropathic pain (CCI model)	CCI + tFUS group (*n*=10); CCI group (*n*=10); sham group (*n*=10)	ACC	21 days, 15mins/day	tFUS with facilitatory effects: A focused transducer (4MHz) was used with an acoustic intensity of 0.95 MPa, DC=10%, PRF=1.5 kHz	Von frey test	3, 10, 17, 20 days, after tFUS treatment	MWT↑ significantly 10, 17, 20 days after the beginning of tFUS treatment	No AEs was induced, assessed by H&E and FJC staining
Zhang et al. 2022 [36]	Mouse model of acute inflammatory pain (Sprague-Dawley male adult rats injected by 50 µl of 3% formalin solution into the rat’s left hindpaw)	tFUS group (*n*=6); control group (*n*=6)	PAG	Single-session for 5 minutes	tFUS with facilitatory effects: F_0_=650 kHz PD = 1 ms PRF=100 Hz ISI=5s DC=10% ISPTA=691mW/cm^2^ MI=0.58	LFP activities in the L5 dorsal horn	Baseline: 5 mins Before stimulation: 30 mins tFUS stimulation： 5mins After stimulation: 5mins	LFP intensity↓ during tFUS stimulation, particularly in the delta, theta, beta and gamma waves; however, once the ultrasound was turned off, the LFP activity gradually returned to its original high state	No significant brain tissue injury was observed, evidenced by histology results. Temperature measurements during tFUS stimulation showed no notable temperature change
Mishra et al. 2023 [37]	Adult macaque monkey	Heat + tFUS group; Heat group (*n*=4 in the crossover design)	Right THAL, specifically the VPL and its surrounding nuclei	tFUS delivered concurrently with heat stimulus administered in an interleaved pattern, with a total of 7 blocks, each lasting 16 seconds.	tFUS with inhibitory effects: F_0_=650kHz PRF=1kHz PD=0.5ms SD=0.5s DC=50% ISI=2s	BOLD fMRI signal evoked by 47.5°C heat stimulation of the left finger in 24 regions of the cortical cortex	During stimulation	Heat-evoked BOLD signal significantly↓ across the brain when tFUS was delivered to VPL concurrently, including the thalamic VPL nuclei along with multiple cortical areas belong to different pain networks.	No tissue damage caused by ultrasound exposure was detected, evidenced by MRI.
Kim et al. 2024 [34]	Mouse models of chronic pain (SCD model, female humanized HbSS-BERK mice, 5-9 months)	Real treatment group (*n*=10); Negative control (*n*=10); sham treatment (*n*=10)	Left S1, IC, THAL	Single-session for 20 minutes	tFUS with inhibitory effects: F_0_=1.5MHz PRF=40Hz PD=0.2ms ISI=4s SD=400ms ISPTA=2.02mW/cm^2^ tFUS with facilitatory effects: PRF=3kHz PD=0.2ms ISI=4s SD=400ms DC=0.8% ISPTA=208.46mW/cm^2^	Hot-plate test; Cold-plate test; Von frey test	At baseline, and at 8mins, 30mins, 60mins after tFUS treatment	Targeting left S1HL (PRF=40Hz): Heat, cold and mechanical hypersensitivity↓ 8 mins after treatment Targeting left S1HL (PRF=3kHz): Heat and mechanical hypersensitivity↑ 8 mins after treatment Targeting left insula (PRF=40Hz): Mechanical and cold hypersensitivity↓ 8 mins; Heat hyperalgesia↓ 8mins, 30mins after treatment, but not 60mins	No AEs on motor function were observed, assessed by rotarod behavioral test No significant changes in tissue structural integrity, iron deposits, or the number of apoptotic cells were noted in histological evaluations.
	SCD model, older female humanized HbSS-BERK mice (10-15 months)	20-min tFUS group (*n*=8); 60-min tFUS group (*n*=7)	Left S1HL	Single session for 20mins or 60mins	tFUS with inhibitory effect: PRF=40Hz PD=0.2ms ISI=4s SD=400ms DC=0.8% ISPTA=2.02mW/cm^2^	Hot-plate test	At baseline, and at 8mins, 30mins after tFUS treatment	60-min tFUS induced Heat hypersensitivity↓ 8 mins after treatment, while that of 20-min tFUS did not differ	
	SCD model, older female humanized HbSS-BERK mice (10-15 months)	S1HL treatment group (*n*=11); Insula treatment group (*n*=10); Sham treatment group (*n*=10)	Left S1HL, IC	Multi-session tFUS: daily 1-hour sessions for 14 days.	PRF=40Hz PD=0.2ms ISI=4s SD=400ms DC=0.8% ISPTA=2.02mW/cm^2^	Hot-plate test	2, 4, 6 hours after tFUS treatment at day 7 and 14	Targeting left S1HL and insula (PRF=40Hz): Heat hypersensitivity↓ last for 2 to 4 hours at the day 14	
	Wild-type mice	Real treatment group (*n*=8M+8F); Sham treatment group	Left S1HL	Single-session tFUS for 10mins	PRF=40Hz PD=0.2ms ISI=4s SD=400ms DC=0.8% ISPTA=2.02mW/cm^2^	Hot-plate test	8 minutes after tFUS treatment	Heat hypersensitivity↓	
Mishra et al. 2025 [38]	Adult macaque monkey	Heat + tFUS group; Heat group (*n*=4 in the crossover design)	Right THAL, specifically the VPL nuclei	tFUS delivered concurrently with heat stimulus administered in an interleaved pattern, with a total of 7 blocks, each lasting 16 seconds.	tFUS with inhibitory effect: F_0_=650kHz PRF=1kHz PD=0.5ms SD=0.5s DC=50% ISI=2s	BOLD fMRI signal evoked by 47.5°C heat stimulation of the left finger	During stimulation	PSC (derived from fMRI BOLD signals) significantly↓ when tFUS was delivered to VPL concurrently	Temperature rise induced by the ultrasound beam does not exceed 2°C and poses no significant thermal risk.
Kim et al. 2025 [35]	Older female humanized SCD mouse model (18-21 months HbSS-BERK)	Multiple circuits group (*n*=10); Sham group (*n*=9)	Left S1HL and IC	Single-session tFUS for 20mins	Single-element transducer (Inhibitory effect): F_0_=500kHz PRF=40Hz PD=0.2ms ISI=4s SD=400ms DC=0.8%	Hot-plate test	10 and 15 min after tFUS treatment	Heat hypersensitivity↓	No AEs on motor function were observed, assessed by rotarod behavioral test
	Older female humanized SCD mouse model (18-21 months HbSS-BERK)	M1 group (*n*=7); S1HL group (*n*=7-8); AI group (*n*=7-8); Sham group (*n*=7-8)	Left S1HL or IC or M1	Single-session tFUS for 20mins	Multi-element transducer (Inhibitory effect): F_0_=1.5MHz PRF=40Hz PD=0.2ms ISI=4s SD=400ms DC=0.8%	Hot-plate test	10 and 15 min after tFUS treatment	M1 vs. Sham: Not significant S1HL vs. Sham: Not significant AI vs. Sham: Not significant	No AEs on motor function were observed, assessed by rotarod behavioral test

*tFUS* Transcranial focused ultrasound stimulation, *F*_0_ Fundamental frequency, *ISPTA* Spatial peak temporal average intensity, *ISPPA* Spatial peak pulse average intensity, *SD* Sonication duration, *PRF* Pulse repetition frequency, *PD* Pulse duration, *DC* Duty cycle, *MI* Mechanical index, *MWT* Mechanical withdrawal threshold; *TWT* Thermal withdrawal threshold, *SCD* Sickle cell disease, *HbSS-BERK* Humanized transgenic homozygous Berkeley sickle mice, *HPT* Heat pain threshold. *S1* Primary somatosensory cortex, *CPT* Cold pain threshold, *S1HL* Hindlimb representation area of the S1, *CCI* Chronic constrictive injury, *ACC* Anterior cingulate cortex, *PAG* periaqueductal gray, *LFP* Local field potential, *THAL* Thalamus, *VPL* Ventral posterior lateral nucleus, *IC* Insula cortex, *PSC* Percentage signal change, *M1* Primary motor cortex

### Risk of bias within included studies

Based on the RoB 2 assessment, five out of six human studies were categorized with a moderate RoB, while one study showed a low RoB (refer to Table S1 and Figures S1-S2 in Supplement 3). For the evaluation of the five animal studies, SYRCLE’s RoB tool was employed. The item distribution was as follows: 0% “no,” suggesting a high RoB; 64.3% “unclear,” pointing to a lack of clarity; and 35.7% “yes,” which corresponds to a low RoB (refer to Table S2 and Figures S3-S4 in Supplement 3 for a comprehensive overview). A summary of the RoB assessments for both human and animal studies can be found in Supplement 3, with Tables S1 and S2 outline the specific criteria for each evaluation instrument and offer a study-specific breakdown.

### Neuromodulation effects of LITUS on pain

**Fig. 2:**
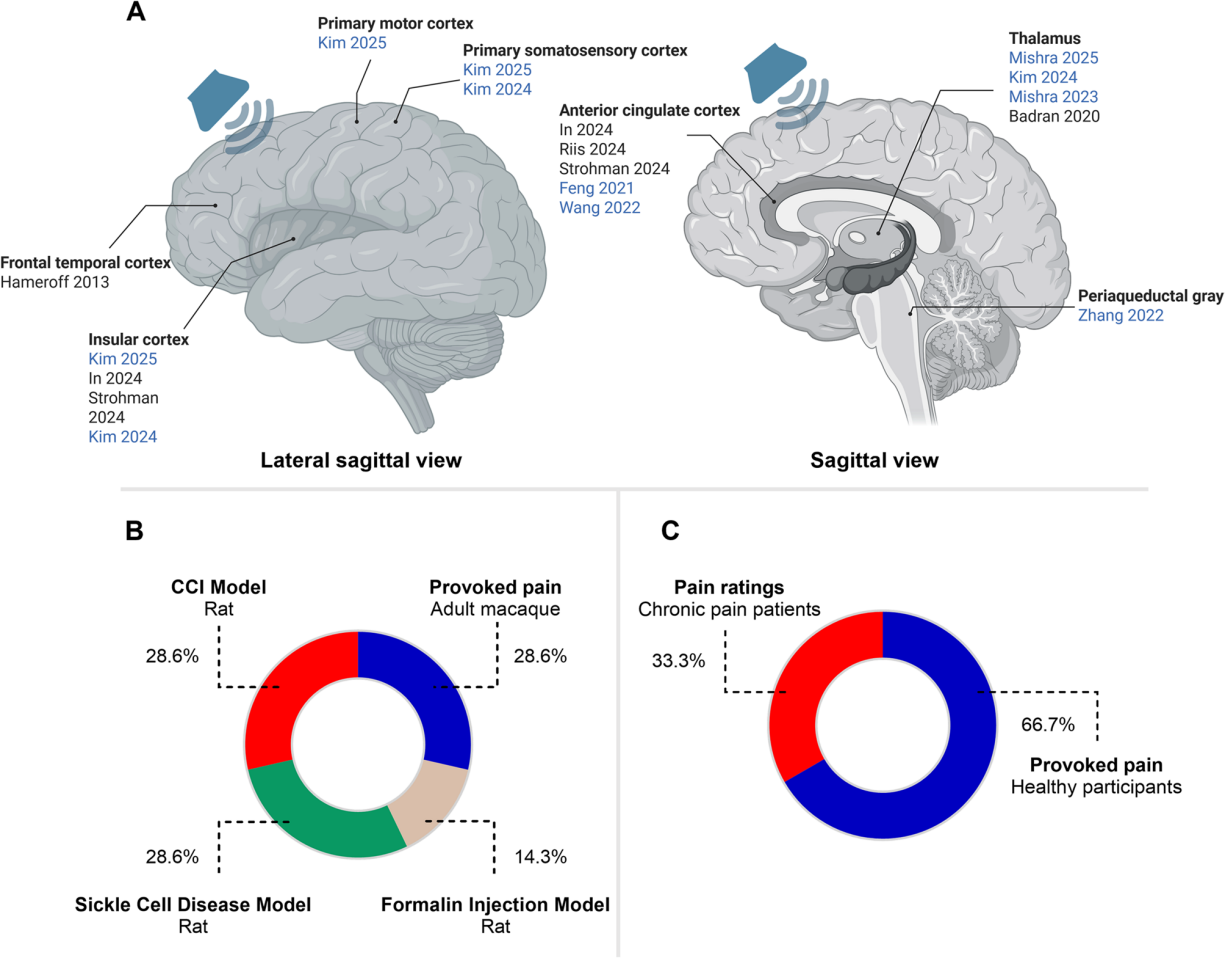
Visualization of existing LITUS targets and subject characteristics in pain neuromodulation. Note:Visualization of existing LITUS targets and subject characteristics in pain neuromodulation. **A** Neuromodulation targets used in existing studies employing LITUS for pain modulation. Studies displayed in blue font represent animal studies and studies in black font represent human studies. **B** Pain models utilized among included animal studies. **C** Participant populations involved among included human studies

In the included research, there is a trend for selecting deep targets such as ACC in 5 studies [28, 29, 31–33], THAL in 4 studies [27, 34, 37, 38], IC in 4 studies [29, 30, 34, 35], the primary somatosensory cortex (S1) in 2 studies [34, 35], the M1 in 1 study [35], the periaqueductal gray (PAG) in 1 study [36], and the frontal-temporal cortex in 1 unfocused LITUS study [22]. One study has initiated attempts to simultaneously target the AI and S1 brain regions [35]. Notably, only one study conducted by Kim and colleagues applied variable DC and PRF to explore the influence of different LITUS parameters on analgesic effects [34]. The visualization of existing TUS Targets and subject Characteristics of included studies was presented in Fig. 2. To better summarize the neuromodulation effects of LITUS on pain, the online and offline effects will be reported separately based on target regions. Out of the 13 studies included, 12 studies demonstrated that LITUS could have a positive and statistically significant impact on pain outcomes.

#### Anterior cingulate cortex

A total of five studies have explored the impact of LITUS on the ACC. Among these, three human studies with inhibitory LITUS were conducted, with one focusing on the subgenual ACC (sACC) and anterior midcingulate cortex (aMCC) [31], and remaining two studies targeting the dorsal ACC (dACC) [28, 29]. Additionally, two animal studies engaged the ACC without verifying specific subregions [32, 33]. Results from four studies underscored the analgesic effectiveness of LITUS measured by numerical rating scale (NRS) [28, 29], Patient-Reported Outcomes Measurement Information System (PROMIS) [31], contact heat-evoked potential (CHEP) amplitude [28], mechanical withdrawal threshold (MWT) [33] and thermal withdrawal threshold (TWT) [32, 33].

In the human study by Riis, individuals with CP underwent 16 trials of 30 s tFUS sessions to evaluate the online analgesic effects. Participants provided verbal feedback on any changes in their pain perception immediately following each session. The study revealed a significant reduction in NRS compared to sham sessions. Moreover, a single 40-minute session of tFUS led to offline improvements in NRS scores and PROMIS measures, which were maintained for over a week post-intervention. While anxiety and depression ratings were also monitored, only a non-significant decreasing trend was observed after intervention [31]. Strohman and colleagues employed tFUS in conjunction with 40 trials of heat stimuli to healthy participants, observing a significant online effect, as evidenced by a reduction in mean NRS scores. Additionally, they recorded a 38.1% decrease in the P2 amplitude of CHEPs in response to noxious stimuli at the Cz electrode. The study also demonstrated improvements in autonomic function, with specific enhancements in the standard deviation of the normal-to-normal intervals (SDNN) and the low-frequency to high-frequency ratio (LF/HF) in response to acute heat pain stimuli [28]. Meanwhile, two animal studies collectively revealed that tFUS to the ACC could alleviate pain in CCI rodents with NP [32, 33]. Feng’s research highlighted a heterogeneous analgesic impact on NP in different stage. Specifically, the effect was online in chronic NP stage and offline in the acute NP stage. To elaborate, the chronic NP model exhibits immediate and sustained alleviation effects after sonication, which is in stark contrast to the acute NP model where the effects are delayed, becoming evident only after two weeks post-surgery. However, Feng’s study demonstrated an inhibitory effect of tFUS on the ACC, as evidenced by a significant decrease in firing frequency within the ACC during and after stimulation. In contrast, Wang’s study showed an activating effect, supported by increased c-fos expression in the ACC. Nevertheless, In’s study did not record significant NRS enhancements in conditioned pain modulation task and temporal summation of pain task following the intervention among healthy participants [29].

#### Thalamus

Four studies have investigated the effects of LITUS on the THAL, uniformly showing inhibitory effects. One study focused on healthy human subjects, targeting the right anterior THAL [27]. The other three studies were animal-based. One focused on modulating THAL in a SCD mouse model [34], while the other two examined the ventral posterolateral nucleus (VPL) within the THAL of healthy monkeys [37, 38]. The outcome measures across these studies included a series of thermal threshold [27], NRS [27], MWT [34], TWT [34], and fMRI BOLD signal [37, 38].

Bardran’s team applied a 20-min tFUS to the right anterior part of THAL among healthy participants [27]. They assessed the offline and online effects of tFUS by measuring thermal thresholds before and after the intervention, and by rating standardized pain immediately after each ultrasound sonication, respectively. Active tFUS significantly attenuated thermal pain thresholds immediately after intervention, whereas no significant changes in sensory or tolerance thresholds was found. For the online effect, there was no significance difference in pain ratings during concurrent pain stimulation. In two animal studies conducted by Mishra et al., tFUS was administered concurrently with nociceptive heat stimuli, resulting in a significant attenuation on the functional Magnetic Resonance Imaging (fMRI) blood oxygen level dependent (BOLD) signal changes evoked by heat noxious stimuli in the targeted THAL nuclei and off-target cortical areas within distinct pain networks [37, 38]. However, a single 20-minute session of tFUS failed to yield any pain-behavioral alterations in the CP model of SCD mice [34].

#### Insula

Four studies have delved into the effects of LITUS on the IC, with a consistent application of inhibitory effects. Two human studies individually targeted the anterior insula (AI) and posterior insula (PI) of healthy participants, while two animal studies focused on the IC of SCD mice. The outcome measures include NRS [29, 30], CHEP amplitude [30], MWT and TWT [34, 35].

In’s human study found that tFUS applied to the PI significantly diminished pain scores in the temporal summation of pain task, a measure indicative of the escalation of pain due to successive noxious inputs, thereby reflecting bottom-up pain processing. Furthermore, tFUS to the PI led to a trend toward attenuation in pain during the conditioned pain modulation task, which assesses the modulatory response to a noxious test stimulus in the context of an ongoing painful conditioning stimulus, suggesting an examination of descending pain inhibitory pathways and top-down pain processing. Conversely, tFUS to AI did not yield significant outcomes [29]. These effects were observed offline. Legon’s study conducted tFUS on healthy participants, which was time-locked to the onset of brief noxious heat stimuli. Concurrently, their pain ratings and electroencephalogram (EEG) data were collected to assess online effects. Both sonication to AI and PI significantly lowers the N1/P1 peak-to-peak CHEP amplitudes. Behavioral results showed that sonication to PI resulted in a reduction in self-reported pain scores and sonication to AI resulted in an improvement in autonomic function [30]. Kim’s animal study demonstrated that a single 20-minute session of tFUS, targeted at the IC with a low PRF of 40 Hz, could reduce cold and mechanical hyperalgesia on the contralateral limb of female sickle cell mice for up to 8 min, and heat hyperalgesia for 30–60 min. Furthermore, a 14 days of daily 1-hour tFUS to IC could extend the therapeutic effect of reducing thermal hyperalgesia to 2–4 h post-intervention [34].

#### Other brain regions

One human study applied unfocused LITUS to the frontal-temporal cortex of CP patients [22]. In animal models, two studies targeted the S1 and M1 in SCD mice model [34, 35], while another study focused on PAG in formalin-induced pain mice [36]. The outcome measures included the NRS [22], MWT [34], TWT [34, 35], and local field potential (LEP) [36].

In the pilot study conducted by Hameroff et al. In 2013, an ultrasound machine in B mode was utilized to generate unfocused LITUS on patients with CP, which resulted in a trend towards reduced pain levels as measured by NRS 40 min after intervention. Moreover, a significant mood improvement was found 10 min and 40 min post exposure [22]. A single 20-minute session of tFUS targeted at the S1 with a low PRF of 40 Hz was found to reduce thermal pain sensitivity and hyperalgesia in wild-type mice and female SCD mice, respectively, yet it failed to ameliorate hyperalgesia in aged female SCD mice [34, 35]. In contrast, sonication with a high PRF of 3 kHz was found to reversely increase thermal and mechanical hyperalgesia. After extending the intervention duration, a single 60-minute session of tFUS with a low PRF of 40 Hz effectively reduced thermal hyperalgesia at 8 min post-intervention, with the effect disappeared by 30 min post-intervention. The above findings might suggest that the dosage of tFUS protocol was insufficient to induce lasting positive changes in pain behavior in the elderly CP mouse model. When the single stimulation protocol was escalated to a long-term intervention, the daily application of 1-hour tFUS sessions over a period of 14 days targeting the S1 significantly mitigated thermal hyperalgesia in aged female SCD mice, with the therapeutic aftereffect persisting for 2 to 4 h post-intervention [34]. According to the latest research by Kim, a single 20-minute session of tFUS stimulation to the M1 did not lead to heat hyperalgesia improvement 10 and 15 min after interventions in aged female SCD mice [35]. Zhang et al. constructed a pain model by injecting formalin into the rat’s hind paw and observed the LFP activities at the L5 dorsal horn and PAG area before, during, and after a 5-minute facilitatory tFUS. During the stimulation, the LFP intensity significantly decreased, indicating a reduction in neural activity caused by painful stimuli. However, the analgesic effect was online but temporary, as the LFP intensity gradually returned to its original state once the stimulation was terminated [36].

#### Multiple targets stimulation

Kim’s team used a single session of 20-minute inhibitory LITUS to simultaneously target the S1 and AI in aged female SCD mice [35]. The intervention employed a single-element transducer at 500 kHz, which is well-suited for covering larger areas, and was driven by a custom-built battery-powered ultrasound analog front end. This approach achieved simultaneous modulation of multiple brain regions to observe the offline neuromodulation effects. Results showed significant improvements in heat hyperalgesia at 10- and 15-minutes post-stimulation compared to baseline and controls.

### Adverse events

To assess the safety of the studies included, we have summarized three key indicators of the FDA-regulated power limits for diagnostic ultrasound: intensity spatial peak pulse average (ISPPA), intensity spatial peak temporal average (ISPTA), and mechanical index (MI). In human studies, all studies reported ISPPA, which are within the safety range (ISPPA ≤ 190 W/cm^2^); at the same time, five (83.3%) reported ISPTA [22, 27–29, 31], with three within the safety range (ISPTA ≤ 720 mW/cm^2^) [22, 27, 31], while Strohman et al.‘s study used an ISPTA of 1.56 W/cm^2^ [28], and In et al.‘s studies were 1.5 W/cm^2^ and 1.62 W/cm^2^ [29], respectively. Four (66.7%) reported MI, all of which were within the safety range (MI ≤ 1.9) [22, 27, 29, 31].

A total of 125 subjects (325 person-times due to crossover design) were enrolled across six human studies investigating the safety and tolerability of the intervention. Four out of six studies using the Report of symptoms questionnaire to observe the presence of symptoms and its severity [28–31]. Riis et al. detected some minor AEs including mild headache, dry mouth, nausea, being resolved within 24 h [31]. Barden et al. reported no AEs throughout their experiment [27]. Strohman et al. noted no moderate or serious AEs, but minor AEs including, but not limited to unusual feelings, tingling, itching, sleepiness, inattentiveness were reported [28]. In and Legon et al. found no severe AEs, with some report sleepiness and anxiousness [29, 30]. Hameroff et al. reported one subject experienced a transient exacerbation of a headache [22].

In the seven included animal studies, five were conducted on rodents [32–36], and two was conducted on monkeys [37, 38]. All rodent studies performed histological analysis and two of them also conducted temperature evaluation [32, 36]. Kim’s studies additionally utilized behavioral tests such as the rotarod to assess motor function [34, 35]. In the non-human primates, Mishra and colleagues applied MRI and temperature evaluation to determine if LITUS caused any damage to brain tissue [37, 38]. All animal studies indicated that LITUS did not result in any AEs. Overall, the safety assessment of the included studies has confirmed that LITUS is safe for pain management, with only minor and transient AEs that resolved quickly, highlighting its potential for clinical use.

## Discussion

In this study, a systematic review of all existing human and animal research utilizing LITUS for pain treatment was presented. We comprehensively summarized the online and offline effects of LITUS on pain based on the targeted brain regions modulated among included studies. Additionally, we assessed the safety of LITUS in pain management by examining AEs and key parameters outlined by the FDA-regulated power limits across all studies. Our study provides integrated evidence for the efficacy of LITUS in the non-invasive management of pain in both clinical and preclinical settings. This includes its application in inducing experimental pain in healthy subjects, treating CP conditions, and its impact in animal models such as the CCI, SCD and provoked pain among non-human primates. These results suggest that LITUS is a promising and safe technique in the field of pain management. Some studies have begun to employ neuroimaging techniques such as EEG and fMRI, as well as physiological monitoring technologies like electrocardiogram and electrodermal response, to elucidate the potential mechanisms underlying the analgesic effects of LITUS. However, a comprehensive synthesis from multiple perspectives is still required to fully understand these mechanisms.

### Overall efficacy and potential influential factors of LITUS on pain

Current research indicates that LITUS targeting various brain regions, including IC, THAL, S1, ACC, and PAG could improve various pain metrics. However, a few trials within the included studies report ineffective outcomes. Based on current evidence, several potential factors might lead to inadequate pain alleviation effect of LITUS.

First, the efficacy of LITUS can be influenced by individual differences. For instance, a single 20-minute LITUS session targeting the S1 reduced pain sensitivity in wild-type rodents and hyperalgesia in female SCD rodents. However, the same protocol was inadequate to achieve pain alleviation in aged ones [34]. This finding suggests that age serves as a critical factor influencing the therapeutic effects of LITUS. The age-related alternations such as decreased neuroplasticity, brain tissue degeneration, and increased neuroinflammation might underlie the decreased analgesic efficacy of LITUS in aged individuals [39]. Based on these findings, other risk factors known to exacerbate pain, including but not limited to sleep quality [40], psychological factors [41], physical activity level [42], and smoking and alcohol behavior [43], could similarly modulate the efficacy of LITUS in pain management. Further studies are warranted to explore how these individual-specific variables influence the therapeutic outcomes of LITUS and to develop personalized treatment strategies.

Second, ultrasound dosage, including stimulation duration and frequency significantly impacts LITUS’s efficacy. Evidence showed that a single 20-minute tFUS session targeting the S1 failed to reduce pain hypersensitivity in female SCD rodents [34, 35]. In contrast, extending the duration of a single session to 60 min induced transient improvement in hyperalgesia. More notably, switching from a single intervention to daily sessions for 14 days resulted in lasting positive changes that could last for several hours. This suggests that insufficient treatment duration or frequency may not trigger analgesic effects, while adequate ultrasound dosage accumulation could be essential for effective pain management.

Third, selecting the optimal brain region, and even specific subregions or nuclei is essential for optimal treatment. Although preliminary evidence has shown that targeting the IC, THAL, S1, ACC, and PAG with LITUS can reduce various pain-related outcomes, it is important to mention that different brain regions play distinct neuroanatomical roles in pain processing. By achieving high spatial resolution and effective deep brain modulation, LITUS has mark a breakthrough in pain neuromodulation research, enabling detailed exploration of specific subregions and nuclei previously challenging to target with conventional NIBS techniques. For instance, evidence from human research has found that LITUS modulation to AI primarily improve pain-related autonomic responses by integrating cognitive, emotional, and interoceptive information related to top-down pain modulation. In contrast, the PI directly receives nociceptive inputs from the spinal cord and primarily processes the sensory-discriminative aspects of pain through a bottom-up mechanism closely related to pain intensity and immediate perception [30, 44]. Therefore, patients with pain conditions dominated by emotional and cognitive aspects may benefit more from AI ultrasound modulation, while those with strong sensory components may respond better to PI ultrasound modulation. Similarly, LITUS to sACC, aMCC, and dACC has proved to have analgesic effects [28, 31]. While these human studies offer preliminary evidence of the potential and feasibility, it is challenging to reach conclusions about how ACC subregions selections affect the efficacy due to the lack of studies that use subregions as variables to explore analgesic effects. Specifically, the sACC modulates pain-related negative affect and emotional memories [45], while the aMCC integrates nociceptive signals with fear-avoidance behaviors, implicating it in threat-contextualized pain modulation [45]. The dACC encodes both pain intensity and affective components while coordinating autonomic responses [46]. Based on established functional profiles of ACC subregions, we can only postulate that patients exhibiting pain phenotypes dominated by affective components (negative affect, fear, catastrophizing) or autonomic dysfunction may show enhanced responsiveness to LITUS modulation of ACC and its subregions. However, this hypothesis necessitates rigorous validation through trials that stratify cohorts based on psychological profiles and physiological markers, thereby aligning intervention targets with individual neurofunctional vulnerabilities. Beyond cortical targets, the thalamus is pivotal in pain processing through segregated lateral and medial systems [47]. The lateral system, including the VPL and ventroposteromedial nuclei, relays sensory-discriminative information from the body and head to somatosensory cortices. Given the VPL’s role as a key relay station for the spinothalamic tract, it has become an initial target for LITUS neuromodulation within THAL [37, 38]. Meanwhile, the medial system, including the paraventricular thalamic nucleus, central medial nucleus, and mediodorsal nucleus, integrates affective-cognitive dimensions by relaying inputs via the parabrachial nucleus to limbic structures. These nuclei mainly contribute to the subjective experience of pain and its associated negative emotions. Although no study has yet employed LITUS specifically targeting nuclei within the medial system, evidence from animal models demonstrates that the regulation of paraventricular thalamic nucleus could alleviate CP [48], visceral pain [49], and comorbid anxiety [50], while central medial nucleus and mediodorsal nucleus are deeply involved in affective pain processing [51]. These existing findings, though limited, indicate that LITUS modulation of the thalamus and its nuclei holds great potential for pain management. Precisely targeting the nuclei in the lateral and medial systems, and determining the optimal parameters matched to the selected nuclei, remain key issues to be addressed in future research. Overall, more research is needed to determine the optimal brain targets for different pain types and patient groups.

Meanwhile, after determining the stimulated brain regions, Riis’s team developed a personalized target selection strategy that dynamically monitored and adjusted the nuclei based on real-time pain feedback from subjects [31]. This is another potential method to enhance the analgesic effect, which highlights the importance of considering individual differences in pain perception and subregions neuroanatomy when optimizing neuromodulation target.

### Hemispheric selection in LITUS pain neuromodulation

As the hemispheric lateralization of brain functions may influence the efficacy of LITUS in pain management, we made a preliminary conclusion of the selection of hemisphere based on current evidence. Among all included studies, those not targeting the ACC consistently select to stimulate the contralateral side to the site of pain presence or provocation, regardless of whether in human or animal studies [22, 27, 29, 30, 34, 35, 37, 38]. This simulation selection strategy is in line with most of the existing evidence, as well as the guidelines for NIBS in pain treatment. likely due to the decussation of pain pathways in the central nervous system (CNS) [7]. However, this does not indicate that all LITUS stimulation targets should be on the side contralateral to the pain. For instance, the TMS guidelines has simultaneously recommended high-frequency TMS over the dorsolateral PFC and low-frequency TMS over the right dorsolateral PFC for depression treatment, indicating that the therapeutic effects may rely on the modulatory effects of the intervention parameters, with the goal of achieving a normal and dynamic balance between the two hemispheres [52]. Thus, the optimal combination of stimulation parameters, targets, and hemispheres of LITUS pain neuromodulation remains to be explored. Regarding to the studies involving the ACC [28, 29, 31–33], all studies did not specify the left or right side of the stimulated regions. In the studies presented by In and Strohman, the Montreal Neurologic Institute coordinates for dACC was reported as (0, 18, 30), indicating that the ultrasound sonication focused on the midline of the brain. In two animal studies presented by Feng and Wang, the transducer was reported to be placed directly above the brain area of ACC (bregma point). By reviewing the schematic diagrams of transcranial ultrasound stimulation target provided in the Riis and Wang’s studies, the effective range of ultrasound stimulation may cover both sides of the cingulate gyrus [31, 33]. Therefore, the optimal hemisphere selection for LITUS targeting the cingulate gyrus remains unclear. Specifically, it is unclear whether left or right hemisphere stimulation is superior, or whether bilateral concurrent stimulation or unilateral stimulation is preferable. Thus, further research data is required to determine the optimal hemisphere selection for LITUS pain neuromodulation.

### LITUS parameters, brain excitability, and pain perception

The integration of LITUS modulation into clinical pain management requires a comprehensive grasp of its facilitatory and inhibitory effects on neural modulation. To date, the factors which impact the modulatory effect of tFUS remains unclear. Currently, some studies suggesting for the critical role of duty cycle (DC) [15, 53], while a few studies start to focus on the significance of PRF [16, 54] in influencing the modulatory effects of LITUS. Due to the interchangeable relationship between PRF and DC through a formula, it is challenging to discern the individual impacts. To decouple the effects of PRF from DC, Yu et al. maintained a constant DC of 60% across groups while varying PRF from 30 Hz to 4500 Hz. They found that groups with a higher PRF still showed enhanced effects on excitatory neurons [55]. This finding may indicate PRF is also a significant parameter in LITUS modulation. To investigate the effects of inhibitory LITUS with the same DC but varying PRF on pain, three groups of female SCD mice were subjected to three different LITUS protocols in Kim’s study (DC = 0.8; G1: PRF = 10 Hz, pulse duration (PD) = 800µs; G2: PRF = 3 kHz, PD = 2.67µs; G3: PRF = 40 Hz, PD = 200µs). Interestingly, only G3 demonstrated significant pain alleviation. The finding suggests that not all inhibitory protocols with low DC to S1 effectively ameliorate pain behavior, implying that additional parameters might be influential factor [34]. To verify this hypothesis, LITUS with varying PRFs of 40 Hz, 300 Hz, 1 kHz, 2 kHz, and 3 kHz were further utilized to assess the influence of PRF changes on pain-related behavioral responses. The researchers discovered that a PRF of 1 kHz served as a critical threshold, beyond which the effects of LITUS shifted from alleviating to exacerbating pain perception. Specifically, the 40 Hz stimulation to S1 significantly elevated the pain threshold, indicative of an analgesic effect, while the 3 kHz stimulation to S1 led to a decrease in pain threshold, suggesting a pronociceptive effect. These findings underscore the importance of PRF in modulating the effects of tFUS on pain [34]. Kim and colleagues’ findings not only align with previous research on the impact of PRF values on modulatory effects but also provide additional insights. First, they have preliminarily determined the critical turning points of PRF that produce different effects, laying the groundwork for future studies to set parameter schemes. Second, a direct causal inference relationship between low and high PRF LITUS, facilitation and inhibition of brain excitability, and the exacerbation and alleviation of pain behavior was demonstrated, rather than relying on indirect speculation. However, it must be noted that the aforementioned findings may only be applicable to the specific brain region and are based on the results of basic experimental research. Therefore, caution must be paid when generalizing these findings to clinical applications and to other target regions or models of different diseases. In the future, it will be essential to explore the potential influence of additional parameters, such as intensity and sonication duration, on the modulatory effects of tFUS for their potential significance [56].

Notably, tFUS parameters may not be the sole factors influencing the modulatory effects. Riis et al. utilized identical tFUS parameters to target the human aMCC and sACC, revealing region-specific modulatory effects [31]. To elaborate, inhibitory effects in the sACC and facilitatory effects in the aMCC were found through fMRI BOLD signals. This suggests that tFUS effects could be both parameter- and region-dependent, potentially due to cellular architecture variations—such as larger cell bodies and a higher glia-to-neuron ratio in the aMCC. These insights highlight the need for upcoming tFUS studies to integrate neuroimaging methods and consider the neurobiological characteristics of targeted brain areas.

### Mechanism

**Fig. 3:**
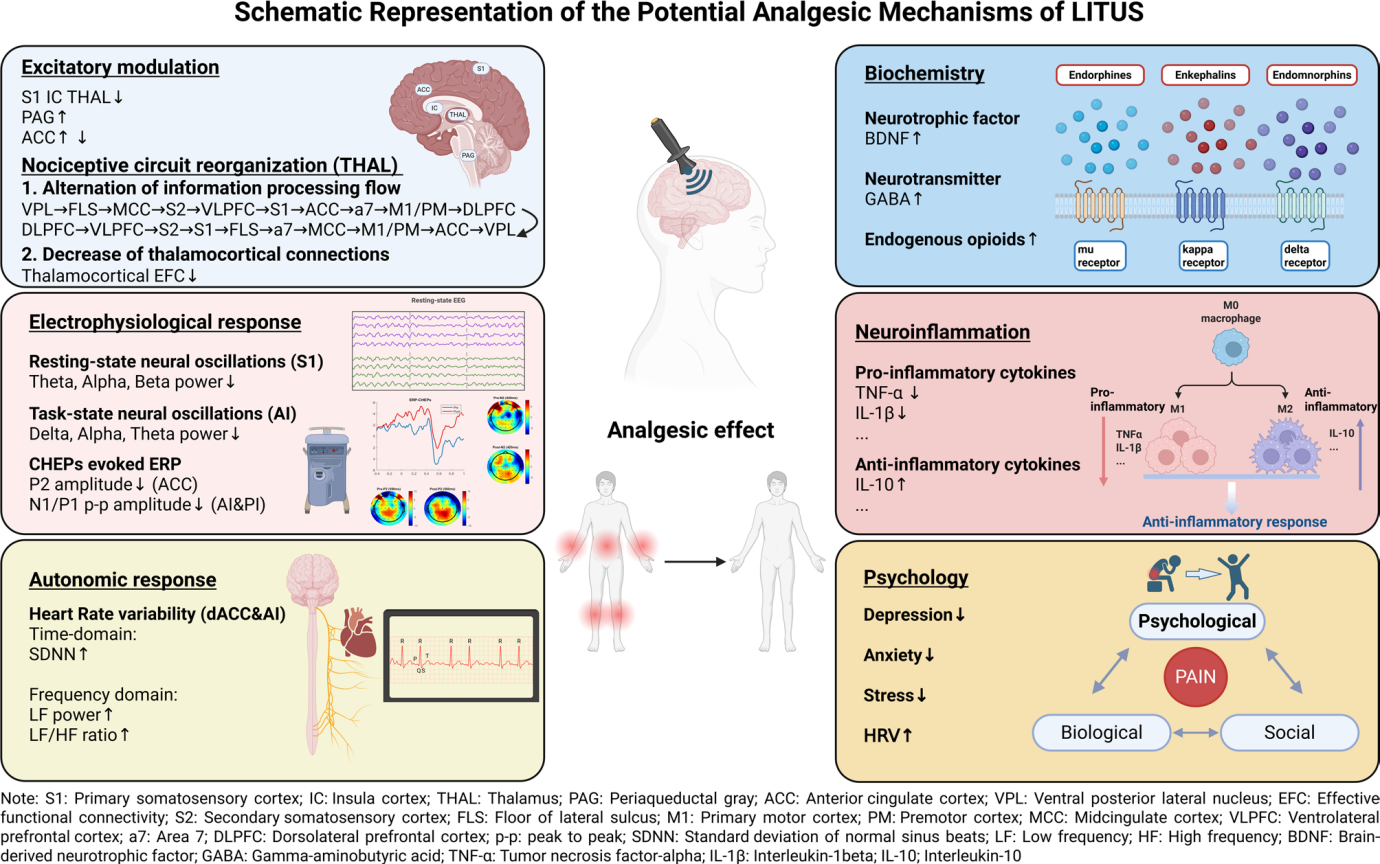
Schematic representation of the potential analgesic mechanisms of LITUS

The potential analgesic mechanisms of LITUS have been summarized based on current evidence, which includes both direct and indirect ones. The schematic representation, viewed from the perspectives of excitatory modulation, nociceptive circuit reorganization, electrophysiological response, autonomic response, biochemistry, neuroinflammation, and psychology, is illustrated in Fig. 3.

#### Mechanism with direct evidence

##### Excitatory modulation and nociceptive circuit reorganization

The pain matrix is a collective term for a specific set of brain regions activated during pain stimulation, including S1, secondary somatosensory cortex (S2), IC, ACC, and THAL [57, 58]. These regions are integral to pain processing and have been consistently targeted in the studies included in this systematic review. Within this matrix, two complementary pathways facilitate pain signal processing: the medial pathway, which projects from the medial THAL to the ACC and IC, managing the affective-motivational dimensions of pain (unpleasantness), and the lateral pathway, which projects from the lateral THAL to the S1, S2, and IC, handling the sensory-discriminative aspects (location and intensity). Previous research has shown that in patients subjected to nociceptive or CP, the S1 [59, 60], IC [61, 62], ACC [63], and THAL [64, 65] exhibit increased abnormal excitability. Furthermore, evidence suggests that applying cathodal transcranial direct current stimulation (tDCS), which generates inhibitory effects, to the S1 and the ACC can effectively reduce pain scores in both healthy subjects and patients experiencing pain [66, 67]. Therefore, LITUS appears to alleviate pain through its modulatory effects by reversing maladaptive changes in the excitability of specific brain regions.

On a broader scale, LITUS not only impacts local brain areas but also instigates a comprehensive reorganization of thalamocortical networks by manipulating nociceptive processing circuits. Mishra and colleagues’ research on primate, leveraging the latencies of fMRI BOLD signal, reveals that inhibitory LITUS targeted at thalamic nuclei significantly alters the information processing flow within the thalamocortical network, resulting in extensive alterations in the brain’s response to nociceptive stimuli and pain signal processing pathways [37]. In response to natural heat stimuli, the VPL nucleus functions as the initial relay point above the spinal cord for processing pain-related heat sensations from the hand, followed by a sequence of activation through floor of lateral sulcus, MCC, S2, Ventrolateral prefrontal cortex (PFC), S1, ACC, area 7, M1, culminating in dorsolateral PFC. However, with LITUS intervention at the VPL, this orderly flow of information processing is markedly disrupted, resulting in altered signal transmission pathways and temporal delays. Notably, the dorsolateral PFC may exhibit an earlier increase in BOLD signals compared to sensory cortices under LITUS stimulation, suggesting a reversal in the direction of information flow from higher-order cortical areas to sensory cortices.

While the latencies of fMRI BOLD signals offer insights into the temporal dynamics of brain activation in response to stimuli, effective functional connectivity (EFC) provides a complementary perspective by emphasizing the directional information flow and causal relationships between brain regions. This delves deeper into the interaction patterns and connectivity strengths that form the basis of the brain’s functional architecture. Within the latest research on primate by Mishra, psychophysiological interaction model was applied to calculate EFC between brain regions [38]. Specifically, they investigated the dynamic connections between the thalamus and the cortex during nociceptive heat processing by comparing conditions with and without LITUS. The results demonstrated that LITUS modulation of the thalamic VPL not only reduced the number of direct thalamocortical connections but also significantly decreased EFC strength in most cortical regions, particularly in sensory, motor, auditory, and higher-order cognitive areas. The Dice similarity coefficient for the EFC networks under two conditions was calculated as 0.7053 and the percentage decrease in correlation power was 17.71% due to LITUS, suggesting that LITUS modulation leads to a weakening of the thalamocortical connections. These findings indicate that the thalamus serves not only as a relay station in pain networks but also dynamically modulates cortical connections. LITUS has a significant impact on the reorganization of the EFC network during pain.

By precisely targeting and modulating key deep brain nuclei associated with pain, LITUS prompts a constructive reorganization of pain networks, shifting them from dysfunctional or maladaptive states toward more efficient and adaptive configurations. This normalization of network activity may represent a central mechanism underlying LITUS’s analgesic effects. The dual capacity of LITUS to both locally modulate the excitability of critical brain regions and globally restructure nociceptive processing circuits positions it as a promising therapeutic tool for clinical pain management.

##### Electrophysiological response

EEG signals, as a non-invasive method, are commonly used for the objective quantification of electrophysiological brain responses to pain [68]. Pain perception is associated with neuronal oscillations and synchronization across various frequencies, and the spectral activity of patients with pain exhibits abnormalities. Compared to healthy individuals, much high-quality evidence has demonstrated that in patients with CP [69] and NP [70, 71], there is an abnormal increase in the amplitude of theta, alpha, beta and gamma frequency oscillations in brain regions related to pain [72]. This finding aligns with Kim’s research, which examined the resting-state neural oscillations in SCD model mice versus WT mice [34]. Following a long-term tFUS intervention for 14 days with each session lasting one hour, it was observed that in older female SCD mice, there was a significant reduction in theta, alpha, and beta frequency oscillatory activities in the resting-state EEG power of the bilateral S1. Notably, this decrease normalized the abnormal oscillatory powers to levels that are nearly indistinguishable from those observed in normal mice, indicating the effectiveness of LITUS on regulating resting state pain-related neural oscillations [34]. Additionally, the early N and P components of EEG brain responses during pain-evoked ERP could robustly encode pain discriminability processing [73–75]. Existing evidence indicates that the P2 wave is the primary component of CHEPs, with its amplitude closely correlated to stimulus intensity and subjective pain perception [76]. Among healthy participants, current evidence has proved that LITUS to ACC attenuates the P2 amplitude of CHEP at Cz electrode [28]. Furthermore, LITUS to IC of lowers the N1/P1 peak-to-peak CHEP amplitudes at Cz electrode [30]. Task-state time frequency analysis revealed that LITUS to IC could reduce the power in delta, alpha and theta frequency during noxious heat stimulus [30]. The collective findings indicate that LITUS may modulate the electrophysiological brain responses to pain, encompassing both the resting-state neural oscillations and the processing of nociceptive information.

##### Autonomic response

Pain is a significant autonomic challenge. CP conditions are frequently associated with dysfunction of the autonomic nervous system, characterized by reduced heart rate variability (HRV) [77]. This HRV dysfunction is indicated by a decrease in SDNN, LF power and LF/HF ratio, which reflect an elevated sympathetic activity or a decreased parasympathetic activity. These alterations in autonomic regulation may attribute to the persistence and exacerbation of pain. Evidence has shown that elevated parasympathetic engagement correlates with enhanced self-regulatory abilities, consequently boosting the capacity for pain inhibition [78]. On a physiological level, LITUS applied to the AI [30] and dACC [28] has been demonstrated to significantly influence HRV among healthy humans, supported by an elevation in SDNN, LF power [79]. This finding indicates a trend toward a normal capacity for dynamic responsiveness to nociceptive inputs within the autonomic system. On a psychological level, pain perception is intricately connected to psychological factors, which play a pivotal role in the bio-psychosocial model [80]. A substantial body of research has demonstrated a correlation between reduced HRV and adverse emotional states, depression, as well as stress [81]. Therefore, LITUS may modulate the overall pain experience by influencing autonomic responses associated with stress and mood [82], thereby altering the cognitive and emotional appraisal of pain. The ability of LITUS to modulate autonomic markers highlights its therapeutic potential in alleviating pain perception by addressing autonomic dysregulation.

#### Other mechanism with indirect evidence

##### Biochemistry

Brain-derived neurotrophic factor (BDNF), a key neurotrophic agent and neuromodulator, is highly expressed in the cerebral cortex and closely associated with maladaptive neuroplastic changes in NP [83]. In the process of pain, BDNF primarily binds to the transmembrane receptor tyrosine kinase receptor B to initiate intracellular signaling cascades in both the peripheral nervous system and CNS [84]. A substantial body of research indicates that NP leads to a downregulation of BDNF expression in various brain regions [85], with pain alleviation being associated with a reversal of these changes in BDNF levels [86, 87]. This correlation underscores the importance of BDNF expression in the manifestation of pain. In animal models of conditions such as depression [88], Parkinson’s disease [18], and attention-deficit/hyperactivity disorder [89], LITUS has been demonstrated to increase BDNF levels in multiple brain areas, suggesting its potential in modulating neurotrophic support, thereby induce analgesic effect.

Gamma-aminobutyric acid (GABA), the principal inhibitory neurotransmitter in the CNS, is indispensable in the regulation of pain perception by mediating inhibitory signals that reduce the excitability of nociceptive neurons [90]. In NP, the inhibitory influence mediated by the GABA system may be diminished, leading to an imbalance between excitation and inhibition in pain pathways. Studies have demonstrated that a reduction in GABAergic activity in the CNS correlates with increased pain severity in patients suffering from CP and NP [90, 91]. Recent findings from a specific study indicate that LITUS can enhance the excitability of GABAergic neurons among schizophrenia patients. Building on this evidence, we speculate that a component of the analgesic effect of LITUS may involve the modulation of GABAergic activity [92].

Endogenous opioids (EOP) provide natural defense against nociceptive input and regulate the downward pain inhibition pathway, thereby influencing our perception and response to pain. While direct evidence linking LITUS to the modulation of the EOP system is yet to be established, our hypothesis that LITUS may partially exert its analgesic effects through this system is supported by previous studies utilizing tDCS [93, 94] and TMS [95]. These studies have demonstrated an upregulation in EOP release within critical deep pain-processing regions, such as the IC, dorsolateral PFC, and ACC, as evidenced by positron emission tomography scan results following NIBS application to the sensorimotor cortex. On the other hand, Zhang’s research, which is included in this review, revealed that LITUS to PAG leads to analgesic effect without further explaining its potential mechanism [36]. The possible explanation is that the PAG is a pivotal central region of the descending pain modulation system, known for its dual role: the dense presence of opioid receptors, including mu, delta, and kappa receptors, and the release of endogenous opioid peptides such as endorphins, enkephalins, and dynorphins [96]. When these opioids bind to the receptors in the PAG, they initiate the activation of descending inhibitory pathways that suppress nociceptive signal transmission at the spinal dorsal horn, thereby alleviating pain. Notably, neuromodulation of the PAG has been rarely realized in studies. However, a study employing deep brain stimulation to PAG in patients with intractable pain showed significant pain relief coincident with positron emission tomography scan indications of EOP release in the PAG [97].

Therefore, we postulate that LITUS could modulate multiple biochemical factors, including BDNF, GABA, and EOP, to indirectly alleviate pain. Whether LITUS could influence other pain-related biochemical factors, including but not limited to 5-hydroxytryptamine, endocannabinoids, and neurotensin, remains a potential direction for future research.

##### Neuroinflammation

Neuroinflammation is a prevalent feature in patients with NP, characterized by excessive inflammation in both the central and peripheral nervous systems, which may play a role in initiating and sustaining CP. Pro-inflammatory and anti-inflammatory cytokines play a pivotal role in the modulation of pain, with their intricate balance being crucial for the pathogenesis of pain conditions [98]. Pro-inflammatory cytokines, such as tumor necrosis factor-alpha (TNF-α) and interleukin-6, interleukin-1beta and Nitric Oxide are known to exacerbate pain by enhancing the transmission of pain signals and inducing central sensitization. The possible mechanisms by which pro-inflammatory cytokines affect pain through neuro-immune/inflammatory pathways include enhancing excitatory currents [99], inhibiting inhibitory currents [100], and promoting the release of excitatory neurotransmitter glutamate via N-Methyl-D-Aspartate receptors [101]. Conversely, anti-inflammatory cytokines, such as interleukin-10 and interleukin-4 are associated with pain reduction by promoting neuroprotection and regeneration, and by counteracting the effects of pro-inflammatory cytokines. Recent rodent studies have revealed that LITUS could downregulate the expression of interleukin-1beta [18] and TNF-α [20], while upregulate the expression of the interleukin-10 within the LITUS targeted area [102]. Although no study has yet investigated the effects of LITUS on inflammatory factors and pain-related indicators concurrently, this hypothesis is supported by research on other NIBS techniques [103]. That is, LITUS may indirectly alleviate pain by modulating the inflammatory response associated with pain. Further research is needed to confirm the effect of LITUS on pain-related neuroinflammation and other inflammatory cytokines.

##### Psychology

Psychological factors, including depression, anxiety, and stress, play a significant role in pain perception and are integral components of the biopsychosocial model of pain [1]. The relationship between pain and depression is bidirectional, with a higher prevalence of depression among patients with CP. Over 50% of patients with CP have depressive symptoms, and patients with depression are twice as likely to develop NP [104, 105]. Anxiety can increase the perception of pain by enhancing expectations and fear responses [106]. Similarly, stress can amplify pain perception, and pain itself is a stressor that affects pain sensitivity through the activation of the hypothalamic-pituitary-adrenal axis and the autonomic nervous system, playing a crucial role in the chronicization of pain [107, 108]. For instance, chronic stress can lead to the sustained activation of the hypothalamic-pituitary-adrenal axis, increasing cortisol release, and the sustained high levels of this hormone are associated with increased pain sensitivity [109]. Moreover, stress may indirectly affect pain perception and emotional responses by impacting emotional regulation areas in the brain, such as the amygdala and PFC [110].

Given the close link between psychological factors and pain perception, LITUS may indirectly exert analgesic effects by modulating emotional states. Direct evidence indicated that LITUS can improve the mood of patients with CP, with a trend towards pain alleviation [22]. Meanwhile, LITUS to brain regions such as dorsolateral PFC [19], medial PFC [20], and right frontal-temporal cortex [111], was found to have positive psychological impact in both humans and animals, such as producing antidepressant effects [19], enhancing vitality and energy levels [22], and reducing anxiety [111]. Furthermore, the impact of LITUS on autonomic responses, which serve as physiological markers of mood and stress, has been previously discussed.

Given the complex interplay between pain and psychological disorder, integrating LITUS with cognitive intervention may serve as a promising pattern to facilitate therapeutic effect. Psychotherapy, such as cognitive behavioral therapy and acceptance and commitment therapy, has been proven to effectively improve pain-related emotional disorders by restructuring patients’ cognition towards pain. Based on the central-peripheral-central rehabilitation theory [112], combining LITUS with psychotherapy may create a closed-loop intervention to further enhance therapeutic effects as existing evidence has shown that combining tDCS with CBT yields better results for pain-related anxiety and fear of movement than either approach alone in patients with chronic low back pain [113].

Consequently, LITUS has the potential to ameliorate psychological conditions such as depression, anxiety, and stress, which may in turn reduce pain perception. Integrating LITUS with psychotherapy might be a potential way to facilitate treatment outcomes for patients with coexisting pain and psychological issues.

### Strengths and limitations

Firstly, this systematic review comprehensively synthesizes the literature on LITUS in pain management, covering both human and animal studies. Based on existing evidence, we have summarized key aspects such as LITUS efficacy in pain relief and its influential factors, targets selection, protocols and safety concerns. This synthesis aims to provide a theoretical foundation that will guide the future application of LITUS research in pain management. Secondly, this article offers a comprehensive review of the potential mechanisms by which LITUS alleviates pain from the aspects of excitatory modulation, nociceptive circuit reorganization, electrophysiological response, autonomic response, biochemistry, neuroinflammation, and psychology. By elucidating these potential mechanisms, we provide insights into how LITUS may interact with the complex pathways involved in pain perception and modulation.

Also, some limitations exist in this study. Firstly, the field of LITUS for pain modulation is relatively nascent, with a considerable body of research focused on animal models and healthy subjects. This presents a gap that must be bridged before the findings in this review can be effectively translated into clinical applications. Secondly, while this review has summarized potential mechanisms by which LITUS may alleviate pain from multiple perspectives, based on both direct and indirect evidence, it is important to acknowledge that due to the current scarcity of research, there may be other mechanisms not yet discussed. Thirdly, despite our systematic aggregation of the analgesic effects of LITUS on pain, the heterogeneity in participants, target sites, and sonication parameters across studies constrains the generalizability of the findings. Consequently, we can only provide descriptive review findings and are precluded from employing meta-analysis methods to quantitatively synthesize and integrate the evidence.

### Future implications

To date, only two studies have specifically targeted CP populations, addressing clinical pathological pain in humans. However, the etiological heterogeneity among the CP patients included in these studies is significant, suggesting that further subgroup analysis may be necessary to facilitate the clinical application in CP patients. The current research status reflects the promising but early stage of LITUS applications in pain management. More research in animals and on experimentally induced pain will provide the foundation for clinically applying LITUS in patients with pathological pain. Excitingly, a preliminary study has begun to focus on patients with cancer-related NP, demonstrating an improvement in pain scores, although it was excluded due to the lack of control group [114]. In the future, LITUS may be applied to a wide range of pain conditions, including but not limited to complex regional pain syndrome, central post-stroke pain, spinal cord injury related NP, migraine and fibromyalgia. Beyond optimizing interventions and exploring alternative targets, Kim’s latest research In 2025 has pioneered the modulation of multiple targets within pain circuits, demonstrating its superiority over single-region modulation in pain management [35]. Thus, future research should focus on facilitating multi-region modulation capabilities of tFUS devices, while exploring combinations of key targets across different pain circuits through high-quality studies to further improve therapeutic outcomes.

## Conclusions

LITUS is an effective and non-invasive technique for pain regulation in both animals and humans, making precise modulation of deep nuclei and circuits possible. Subjects with pain associated risk factors, insufficient LITUS dosage, suboptimal LITUS protocol and target selection mismatched to specific pain conditions, may lead to reduced analgesic effects of LITUS. Preliminary evidence has elucidated the direct relationship between LITUS parameters, brain region responses, and pain behavior. The analgesic effects of LITUS may be attributed to a series of comprehensive mechanisms. There is an urgent need for more high-quality research to facilitate the clinical application of LITUS in pain patients and to uncover the mechanisms underlying its effects.

## Supplementary Information

Supplementary Material 1: Searching Strategy.

Supplementary Material 2: Reasons for exclusion.

Supplementary Material 3: Table S1 Full breakdown of ROB assessment for the included human studies using the Cochrane Risk of Bias 2 tool. Supplementary Table S2 Full breakdown of ROB assessment for the included animal studies using the Systematic Review Center for Laboratory Animal Experimentation Risk of Bias tool. Figure S1 Graphical representation of ROB assessment for included human studies using the Cochrane Risk of Bias 2 tool. Supplementary Figure S2 Percentage distribution of ROB assessment for included human studies using the Cochrane Risk of Bias 2 tool. Supplementary Figure S3 Graphical representation of ROB assessment for included animal studies using the Systematic Review Center for Laboratory Animal Experimentation Risk of Bias tool. Supplementary Figure S4 Percentage distribution of ROB assessment for included animal studies using the Systematic Review Center for Laboratory Animal Experimentation Risk of Bias tool.

## Data Availability

No datasets were generated or analysed during the current study.

## Abbreviations

AEs: Adverse events
ACC: Anterior cingulate cortex
aMCC: Anterior midcingulate cortex
AI: Anterior insula
BDNF: Brain-derived neurotrophic factor
BOLD: Blood oxygen level dependent
CCI: Chronic constriction injury
CHEP: Contact heat-evoked potential
CNS: Central nervous system
CP: Chronic pain
dACC: dorsal ACC
DC: Duty cycle
EEG: Electroencephalogram
EFC: Effective functional connectivity
EOP: Endogenous opioids
fMRI: Functional magnetic resonance imaging
GABA: Gamma-aminobutyric acid
HRV: Heart rate variability
IC: Insular cortex
LF: low frequency
HF: High frequency
LFP: Local field potential
M1: Primary motor cortex
MWT: Mechanical withdrawal threshold
NIBS: Non-invasive brain stimulation
NP: Neuropathic pain
NRS: Numerical rating scale
PAG: Periaqueductal gray
PFC: Prefrontal cortex
PRF: Pulse repetition frequency
PI: Posterior insula
PROMIS: Patient-reported outcomes measurement information system
sACC: Subgenual ACC
SCD: Sickle cell disease
SDNN: Standard deviation of the normal-to-normal intervals
S1: Primary somatosensory cortex
tDCS: Transcranial direct current stimulation
tES: Transcranial electrical stimulation
THAL: Thalamus
tFUS: Transcranial focused ultrasound
TMS: Transcranial magnetic stimulation
TNF-α: Tumor necrosis factor-alpha
TUS: Transcranial ultrasound
TWT: Thermal withdrawal threshold
VPL: Ventral posterolateral nucleus
