# Supplementary material for: The efficacy and mechanisms of low-intensity transcranial ultrasound stimulation on pain: a systematic review of human and animal studies
Source: J Headache Pain. 2025 Jul 22;26(1):166. doi: 10.1186/s10194-025-02096-y (PMC12281706; doi:10.1186/s10194-025-02096-y)
Supplement: Supplementary file 1 — Supplementary Material 1: Searching Strategy. [file 10194_2025_2096_MOESM1_ESM.docx]

1. Search strategy of MEDLINE through PubMed

#1 "low intensity transcranial ultrasound"

#2 "low-intensity transcranial ultrasound"

#3 "transcranial focused ultrasound"

#4 "transcranial unfocused ultrasound"

#5 "transcranial pulse stimulation"

#6 "transcranial ultrasound"

#7 "low intensity transcranial focused ultrasound"

#8 "low-intensity transcranial focused ultrasound"

#9 "low intensity focused ultrasound"

#10 "low-intensity focused ultrasound"

#11 #1 OR #2 OR #3 OR #4 OR #5 OR #6 OR #7 OR #8 OR #9 OR #10

Number:1439

#12 "focused ultrasound"

#13 transcranial

#14 ultrasound

#15 #12 AND #13

Number:813

#16 #13 AND #14

Number:15718

#17 #11 OR #15 OR #16

Number:15994

#18 pain

Number:1120745

#19 #17 AND #18

Number:558

Filters Results by year: from Inception to 31 Mar 2025

Language: English

Source: MEDLINE

1. Search strategy of WOS through Web of Science

#1 "low intensity transcranial ultrasound"

#2 "low-intensity transcranial ultrasound"

#3 "transcranial focused ultrasound"

#4 "transcranial unfocused ultrasound"

#5 "transcranial pulse stimulation"

#6 "transcranial ultrasound"

#7 "low intensity transcranial focused ultrasound"

#8 "low-intensity transcranial focused ultrasound"

#9 "low intensity focused ultrasound"

#10 "low-intensity focused ultrasound"

#11 #1 OR #2 OR #3 OR #4 OR #5 OR #6 OR #7 OR #8 OR #9 OR #10

Number:1611

#12 "focused ultrasound"

#13 transcranial

#14 ultrasound

#15 #12 AND #13

Number:1065

#16 #13 AND #14

Number: 4744

#17 #11 OR #15 OR #16

Number: 5081

#18 pain

Number: 703055

#19 #17 AND #18

Number:134

Filters Results by year: from 1900-01-01 to 31 Mar 2025

Language: English

Source: WOS

1. Search strategy of CINAHL through EBSCOHost

#1 "low intensity transcranial ultrasound"

#2 "low-intensity transcranial ultrasound"

#3 "transcranial focused ultrasound"

#4 "transcranial unfocused ultrasound"

#5 "transcranial pulse stimulation"

#6 "transcranial ultrasound"

#7 "low intensity transcranial focused ultrasound"

#8 "low-intensity transcranial focused ultrasound"

#9 "low intensity focused ultrasound"

#10 "low-intensity focused ultrasound"

#11 #1 OR #2 OR #3 OR #4 OR #5 OR #6 OR #7 OR #8 OR #9 OR #10

Number: 187

#12 "focused ultrasound"

#13 transcranial

#14 ultrasound

#15 #12 AND #13

Number: 64

#16 #13 AND #14

Number: 763

#17 #11 OR #15 OR #16

Number: 787

#18 pain

Number: 299586

#19 #17 AND #18

Number: 12

Filters Results by year: from Inception to 31 Mar 2025

Language: English

Source: EBSCOHost

1. Search strategy of Embase through Embase

#1 "low intensity transcranial ultrasound"

#2 "low-intensity transcranial ultrasound"

#3 "transcranial focused ultrasound"

#4 "transcranial unfocused ultrasound"

#5 "transcranial pulse stimulation"

#6 "transcranial ultrasound"

#7 "low intensity transcranial focused ultrasound"

#8 "low-intensity transcranial focused ultrasound"

#9 "low intensity focused ultrasound"

#10 "low-intensity focused ultrasound"

#11 #1 OR #2 OR #3 OR #4 OR #5 OR #6 OR #7 OR #8 OR #9 OR #10

Number: 2129

#12 "focused ultrasound"

#13 transcranial

#14 ultrasound

#15 #12 AND #13

Number: 1242

#16 #13 AND #14

Number: 8084

#17 #11 OR #15 OR #16

Number: 8544

#18 pain

Number: 1708816

#19 #17 AND #18

Number: 430

Filters Results by year: from Inception to 31 Mar 2025

Language: English

Source: Embase

1. Search strategy of CENTRAL through Cochrane Library

#1 "low intensity transcranial ultrasound"

#2 "low-intensity transcranial ultrasound"

#3 "transcranial focused ultrasound"

#4 "transcranial unfocused ultrasound"

#5 "transcranial pulse stimulation"

#6 "transcranial ultrasound"

#7 "low intensity transcranial focused ultrasound"

#8 "low-intensity transcranial focused ultrasound"

#9 "low intensity focused ultrasound"

#10 "low-intensity focused ultrasound"

#11 #1 OR #2 OR #3 OR #4 OR #5 OR #6 OR #7 OR #8 OR #9 OR #10

Number: 225

#12 "focused ultrasound"

#13 transcranial

#14 ultrasound

#15 #12 AND #13

Number: 75

#16 #13 AND #14

Number: 677

#17 #11 OR #15 OR #16

Number: 744

#18 pain

Number: 273094

#19 #17 AND #18

Number: 90

Filters Results by year: from Inception to 31 Mar 2025

Language: English

Source: CENTRAL
