# Supplementary material for: The efficacy and mechanisms of low-intensity transcranial ultrasound stimulation on pain: a systematic review of human and animal studies
Source: J Headache Pain. 2025 Jul 22;26(1):166. doi: 10.1186/s10194-025-02096-y (PMC12281706; doi:10.1186/s10194-025-02096-y)
Supplement: Supplementary file 2 — Supplementary Material 2: Reasons for exclusion. [file 10194_2025_2096_MOESM2_ESM.docx]

**Excluded human studies after full-text review (n=23); Included (n=6)**

**Reason for exclusion 1:** Peripheral ultrasound instead of transcranial ultrasound (n=4)

(Bishay et al., 2024; Heng, Ping, & Xiaodi, 2021; Qin et al., 2024; Wei et al., 2024)

**Reason for exclusion 2:** Duplicate or preprint (n=10)

(Badran et al., 2020; Badran, Caulfield, Stomberg-Firestein, Summers, Dowdle, Savoca, Li, Austelle, Short, Borckardt, & et al., 2022; Badran, Caulfield, Stomberg-Firestein, Summers, Dowdle, Savoca, Li, Austelle, Short, Borckardt, Spivak, et al., 2022; In, Strohman, Payne, & Legon, 2024a, 2024b; Legon, Strohman, In, Stebbins, & Payne, 2023; Strohman, Isaac, et al., 2024a, 2024b; Strohman, Payne, In, Stebbins, & Legon, 2023; Strohman, Payne, et al., 2024)

**Reason for exclusion 3:** Abstract or protocol (n=3)

(Bao, Byraju, Patel, Hellman, Neubauer, Burdette, Mahoney, et al., 2022; Elias, Moosa, & Liu, 2022; jRCTs, 2023)

**Reason for exclusion 4:** No controlled group (n=4)

(Jeanmonod et al., 2012; Martin, Jeanmonod, Morel, Zadicario, & Werner, 2009; Patel et al., 2021; Shin, Son, & Kim, 2023)

**Reason for exclusion 5:** Without pain-related outcome measures (n=2)

(Jones et al., 2020; Moser, Zadicario, Schiff, & Jeanmonod, 2013)

**Excluded animal studies after full-text review (n=20); Included (n=7)**

**Reason for exclusion 1：**Peripheral ultrasound instead of transcranial ultrasound (n=11)

(Bao, Byraju, Patel, Hellman, Neubauer, Burdette, Rafferty, et al., 2022; Hellman, Clum, Maietta, Srikanthan, Patel, Panse, Neubauer, et al., 2021; Hellman, Clum, Maietta, Srikanthan, Patel, Panse, Zimmerman, et al., 2021; A. Hellman, T. Maietta, K. Byraju, Y. Linda Park, et al., 2020; A. Hellman, T. Maietta, K. Byraju, Y. L. Park, et al., 2020; Hellman, Maietta, et al., 2021; Liang et al., 2020; Liao, Wang, Chen, Liu, & Ao, 2021; Lin et al., 2025; Liss et al., 2022; Pilitsis et al., 2020)

**Reason for exclusion 2:** Duplicate or preprint (n=5)

(Abigail Hellman et al., 2020; M. G. Kim et al., 2022; Mishra et al., 2023; Wang et al., 2022; Zhang et al., 2022)

**Reason for exclusion 3:** Abstract or protocol (n=2)

(Min Gon Kim et al., 2022; Mourad, 2024)

**Reason for exclusion 4：**Without pain-related outcome measures (n=2)

(Kim, Lee, Bohlke, Yoon, & Yoo, 2021; Todd et al., 2019)

**References**

Badran, B. W., Caulfield, K. A., Stomberg-Firestein, S., Summers, P. M., Dowdle, L. T., Savoca, M., . . . et al. (2020). Sonication of the anterior thalamus with MRI-Guided transcranial focused ultrasound (tFUS) alters pain thresholds in healthy adults: a double-blind, sham-controlled study. *Brain Stimul, 13*(6), 1805‐1812. doi:10.1016/j.brs.2020.10.007

Badran, B. W., Caulfield, K. A., Stomberg-Firestein, S., Summers, P. M., Dowdle, L. T., Savoca, M., . . . et al. (2022). Sonication of the Anterior Thalamus With MRI-Guided Transcranial Focused Ultrasound (tFUS) Alters Pain Thresholds in Healthy Adults: a Double-Blind, Sham-Controlled Study. *Focus (United States), 20*(1), 90‐99. doi:10.1176/appi.focus.20109

Badran, B. W., Caulfield, K. A., Stomberg-Firestein, S., Summers, P. M., Dowdle, L. T., Savoca, M., . . . George, M. S. (2022). Sonication of the Anterior Thalamus With MRI-Guided Transcranial Focused Ultrasound (tFUS) Alters Pain Thresholds in Healthy Adults: A Double-Blind, Sham-Controlled Study. *Focus (Am Psychiatr Publ), 20*(1), 90-99. doi:10.1176/appi.focus.20109

Bao, J., Byraju, K., Patel, V., Hellman, A., Neubauer, P., Burdette, C., . . . Pilitsis, J. (2022). ID:16372 Effects of Low-Intensity Focused Ultrasound on Neuronal Activity in Pain Processing Regions of the Brain. *Neuromodulation, 25*(5), S79. doi:10.1016/j.neurom.2022.02.100

Bao, J., Byraju, K., Patel, V. J., Hellman, A., Neubauer, P., Burdette, C., . . . Pilitsis, J. G. (2022). The effects of low intensity focused ultrasound on neuronal activity in pain processing regions in a rodent model of common peroneal nerve injury. *Neurosci Lett, 789*, 136882. doi:10.1016/j.neulet.2022.136882

Bishay, A. A. E. D., Swenson, A. J., Spivak, N. M., Schafer, S., Bych, B. P., Gilles, S. D., . . . Bystritsky, A. (2024). Preliminary Examination of the Effects of Focused Ultrasound on Living Skin and Temperature at the Skin-Transducer Interface. *Bioengineering (Basel), 11*(11). doi:10.3390/bioengineering11111126

Elias, W. J., Moosa, S., & Liu, C. C. (2022). Focused Ultrasound Mesencephalotomy for Head and Neck Cancer Pain. *Stereotact Funct Neurosurg, 100*, 6-7. doi:10.1159/000528074

Hellman, A., Clum, A., Maietta, T., Srikanthan, A., Patel, V., Panse, D., . . . Pilitsis, J. (2021). Effects of External Focused Ultrasound on Inflammatory Markers in Common Peroneal Nerve Injury. *Neuromodulation, 24*(4), e244. doi:10.1111/ner.13385

Hellman, A., Clum, A., Maietta, T., Srikanthan, A., Patel, V., Panse, D., . . . Pilitsis, J. G. (2021). Effects of external low intensity focused ultrasound on inflammatory markers in neuropathic pain. *Neurosci Lett, 757*, 135977. doi:10.1016/j.neulet.2021.135977

Hellman, A., Maietta, T., Byraju, K., Linda Park, Y., Shao, M., Liss, A., . . . Pilitsis, J. G. (2020). Low Intensity Focused Ultrasound Modulation of Vincristine Induced Neuropathy. *Neuroscience, 430*, 82-93. doi:10.1016/j.neuroscience.2020.01.021

Hellman, A., Maietta, T., Byraju, K., Park, Y. L., Liss, A., Prabhala, T., . . . Pilitsis, J. (2020). Effects of external low intensity focused ultrasound on electrophysiological changes in vivo in a rodent model of common peroneal nerve injury. *Neuroscience, 429*, 264-272. doi:10.1016/j.neuroscience.2020.01.016

Hellman, A., Maietta, T., Byraju, K., Park, Y. L., Shao, M., Liss, A., . . . Pilitsis, J. G. (2020). Low Intensity Focused Ultrasound Modulation of Vincristine Induced Neuropathy. *Neuroscience, 430*, 82-93. doi:10.1016/j.neuroscience.2020.01.021

Hellman, A., Maietta, T., Clum, A., Byraju, K., Raviv, N., Staudt, M. D., . . . Pilitsis, J. G. (2021). Pilot study on the effects of low intensity focused ultrasound in a swine model of neuropathic pain. *J Neurosurg, 135*(5), 1508-1515. doi:10.3171/2020.9.Jns202962

Heng, Y., Ping, Z., & Xiaodi, L. (2021). Low-intensity focused ultrasound and low-intensity pulsed ultrasound for mild traumatic knee osteoarthritis: Differences in pain and function. *Chinese Journal of Tissue Engineering Research, 25*(26), 4101-4105. doi:10.12307/2021.105

In, A., Strohman, A., Payne, B., & Legon, W. (2024a). Low-intensity focused ultrasound to the insula and dorsal anterior cingulate has site-specific and pressure dependent effects on pain during measures of central sensitization. In.

In, A., Strohman, A., Payne, B., & Legon, W. (2024b). Low-intensity focused ultrasound to the posterior insula reduces temporal summation of pain. *Brain Stimul, 17*(4), 911-924. doi:10.1016/j.brs.2024.07.020

Jeanmonod, D., Werner, B., Morel, A., Michels, L., Zadicario, E., Schiff, G., & Martin, E. (2012). Transcranial magnetic resonance imaging-guided focused ultrasound: noninvasive central lateral thalamotomy for chronic neuropathic pain. *Neurosurg Focus, 32*(1), E1. doi:10.3171/2011.10.Focus11248

Jones, R. M., Kamps, S., Huang, Y., Scantlebury, N., Lipsman, N., Schwartz, M. L., & Hynynen, K. (2020). Accumulated thermal dose in MRI-guided focused ultrasound for essential tremor: repeated sonications with low focal temperatures. *J Neurosurg, 132*(6), 1802-1809. doi:10.3171/2019.2.Jns182995

jRCTs, J. (2023). Perceptual modification using low-intensity transcranial focused ultrasound for neuropathic pain. [*https://trialsearch.who.int/Trial2.aspx?TrialID=JPRN-jRCTs052230116*](https://trialsearch.who.int/Trial2.aspx?TrialID=JPRN-jRCTs052230116).

Kim, H.-C., Lee, W., Bohlke, M., Yoon, K., & Yoo, S.-S. (2021). Focused ultrasound enhances the anesthetic effects of topical lidocaine in rats. *BMC ANESTHESIOLOGY, 21*(1). doi:10.1186/s12871-021-01381-y

Kim, M. G., Yu, K., Argueta, D. A., Kiven, S. B., Ni, Y., Gupta, K., & He, B. (2022). Modulation of Pain-Associated Behaviors in Sickle Mice Using Low-Intensity Transcranial Focused Ultrasound. *Blood, 140*. doi:10.1182/blood-2022-160217

Kim, M. G., Yu, K., Yeh, C. Y., Fouda, R., Argueta, D., Kiven, S., . . . He, B. (2022). Low-intensity transcranial focused ultrasound suppresses pain by modulating pain processing brain circuits. In.

Legon, W., Strohman, A., In, A., Stebbins, K., & Payne, B. (2023). Non-invasive neuromodulation of sub-regions of the human insula differentially affect pain processing and heart-rate variability. In.

Liang, D., Chen, J., Zhou, W., Chen, J., Chen, W., & Wang, Y. (2020). Alleviation Effects and Mechanisms of Low-intensity Focused Ultrasound on Pain Triggered by Soft Tissue Injury. *J Ultrasound Med, 39*(5), 997-1005. doi:10.1002/jum.15185

Liao, Y. H., Wang, B., Chen, M. X., Liu, Y., & Ao, L. J. (2021). LIFU Alleviates Neuropathic Pain by Improving the KCC(2) Expression and Inhibiting the CaMKIV-KCC(2) Pathway in the L4-L5 Section of the Spinal Cord. *Neural Plast, 2021*, 6659668. doi:10.1155/2021/6659668

Lin, Y.-T., Chen, K.-T., Hsu, C.-C., Liu, H.-L., Jiang, Y.-T., Ho, C.-W., . . . Hsu, P.-H. (2025). Stimulation of dorsal root ganglion with low-intensity focused ultrasound ameliorates pain responses through the GABA inhibitory pathway. *Life Sci, 361*, 123323. doi:10.1016/j.lfs.2024.123323

Liss, A., Hellman, A., Patel, V. J., Maietta, T., Byraju, K., Trowbridge, R., . . . Pilitsis, J. G. (2022). Low Intensity Focused Ultrasound Increases Duration of Anti-Nociceptive Responses in Female Common Peroneal Nerve Injury Rats. *Neuromodulation, 25*(4), 504-510. doi:10.1111/ner.13531

Martin, E., Jeanmonod, D., Morel, A., Zadicario, E., & Werner, B. (2009). High-intensity focused ultrasound for noninvasive functional neurosurgery. *Ann Neurol, 66*(6), 858-861. doi:10.1002/ana.21801

Mishra, A., Yang, P.-F., Manuel, T. J., Newton, A. T., Phipps, M. A., Luo, H., . . . Chen, L. M. (2023). Disrupting nociceptive information processing flow through transcranial focused ultrasound neuromodulation of thalamic nuclei. *Brain Stimul, 16*(5), 1430-1444. doi:10.1016/j.brs.2023.09.013

Moser, D., Zadicario, E., Schiff, G., & Jeanmonod, D. (2013). MR-guided focused ultrasound technique in functional neurosurgery: targeting accuracy. *J Ther Ultrasound, 1*, 3. doi:10.1186/2050-5736-1-3

Mourad, P. D. (2024). Lowering pain with LIFU. *Blood, 144*(10), 1035-1036. doi:10.1182/blood.2024025937

Patel, A. A., Zhukosvky, M., Sidharthan, S., Jotwani, R., Rakesh, N., & Gulati, A. (2021). Preliminary effects of low-intensity focused ultrasound treatment program for cancer-related neuropathic pain. *Pain Manag, 11*(5), 613-621. doi:10.2217/pmt-2020-0099

Pilitsis, J., Hellman, A., Maietta, T., Clum, A., Byraju, K., Jeannotte, E., . . . Qian, J. (2020). Effects of external low intensity focused ultrasound in swine model of common peroneal nerve injury. *Neuromodulation, 23*(3), e152. doi:10.1111/ner.13133

Qin, L., Dou, M., Niu, L., Huang, L., Li, F., Bao, S., . . . Geng, Y. (2024). Low-Intensity Focused Ultrasound Stimulation on Fingertip Can Evoke Fine Tactile Sensations and Different Local Hemodynamic Responses. *IEEE Trans Neural Syst Rehabil Eng, PP*. doi:10.1109/TNSRE.2024.3493925

Shin, D. H., Son, S., & Kim, E. Y. (2023). Low-Energy Transcranial Navigation-Guided Focused Ultrasound for Neuropathic Pain: An Exploratory Study. *Brain Sci, 13*(10). doi:10.3390/brainsci13101433

Strohman, A., Isaac, G., Payne, B., Verdonk, C., Khalsa, S. S., & Legon, W. (2024a). Low-intensity focused ultrasound to the human insular cortex differentially modulates the heartbeat-evoked potential: a proof-of-concept study. In.

Strohman, A., Isaac, G., Payne, B., Verdonk, C., Khalsa, S. S., & Legon, W. (2024b). Low-intensity focused ultrasound to the human insular cortex differentially modulates the heartbeat-evoked potential: a proof-of-concept study. *bioRxiv*. doi:10.1101/2024.03.08.584152

Strohman, A., Payne, B., In, A., Stebbins, K., & Legon, W. (2023). Non-invasive modulation of the human dorsal anterior cingulate attenuates acute pain perception and homeostatic cardiovascular responses. In.

Strohman, A., Payne, B., Legon, W., In, A., Strohman, A., Legon, W., . . . Legon, W. (2024). Low-intensity focused ultrasound to the posterior insula reduces temporal summation of pain. *Brain Stimul, 17*(4), 911-924. doi:10.1016/j.brs.2024.07.020

Todd, N., Zhang, Y., Power, C., Becerra, L., Borsook, D., Livingstone, M., & McDannold, N. (2019). Modulation of brain function by targeted delivery of GABA through the disrupted blood-brain barrier. *Neuroimage, 189*, 267-275. doi:10.1016/j.neuroimage.2019.01.037

Wang, B., Chen, M.-X., Chen, S.-C., Feng, X.-J., Liao, Y.-H., Zhao, Y.-X., . . . Ao, L.-J. (2022). Low-Intensity Focused Ultrasound Alleviates Chronic Neuropathic Pain-Induced Allodynia by Inhibiting Neuroplasticity in the Anterior Cingulate Cortex. *Neural Plast, 2022*. doi:10.1155/2022/6472475

Wei, D., Yue, J., Meng, J., Gao, J., Yang, L., Niu, X., & Wang, Z. (2024). Preliminary study of the effect of low-intensity focused ultrasound on postpartum uterine involution and breast pain in puerperal women: a randomised controlled trial. *Sci Rep, 14*(1), 658. doi:10.1038/s41598-024-51328-9

Zhang, T., Wang, Z., Liang, H., Wu, Z., Li, J., Ou-Yang, J., . . . Zhu, B. (2022). Transcranial Focused Ultrasound Stimulation of Periaqueductal Gray for Analgesia. *IEEE TRANSACTIONS ON BIOMEDICAL ENGINEERING, 69*(10), 3155-3162. doi:10.1109/TBME.2022.3162073
