# Supplementary material for: The efficacy and mechanisms of low-intensity transcranial ultrasound stimulation on pain: a systematic review of human and animal studies
Source: J Headache Pain. 2025 Jul 22;26(1):166. doi: 10.1186/s10194-025-02096-y (PMC12281706; doi:10.1186/s10194-025-02096-y)

**Supplementary Table S1** Full breakdown of ROB assessment for the included human studies using the Cochrane Risk of Bias 2 tool

| **Author, Year** | **D1** | **DS** | **D2** | **D3** | **D4** | **D5** | **Overall** |
| --- | --- | --- | --- | --- | --- | --- | --- |
| **Hameroff 2013** | Some concerns | Some concerns | Low risk | Low risk | Low risk | Some concerns | Some concerns |
| **Badran**  **2020** | Some concerns | Some concerns | Low risk | Low risk | Low risk | Low risk | Some concerns |
| **Strohman 2024** | Low risk | Low risk | Some concerns | Low risk | Low risk | Some concerns | Some concerns |
| **Riis**  **2024** | Low risk | Low risk | Low risk | Low risk | Low risk | Low risk | Low risk |
| **In**  **2024** | Low risk | Low risk | Some concerns | Low risk | Some concerns | Some concerns | Some concerns |
| **Legon**  **2024** | Low risk | Low risk | Some concerns | Low risk | Low risk | Low risk | Some concerns |

**Domains for the Cochrane Rob of Bias tool 2:** D1: risk of bias arising from the randomization process; DS: risk of bias arising from period and carryover effects (applicable for crossover trials); D2: risk of bias due to deviations from the intended interventions; D3: risk of bias due to missing outcome data; D4: risk of bias in measurement of the outcome; D5: risk of bias in selection of the reported result.

**Criteria for the overall risk-of-bias judgement:** “Low risk”: the study is judged to be low risk of bias for all domains for this result; “Some concerns”: The study is judged to raise some concerns in at least one domain for this result, but not at high risk of bias for any domain; “High risk”: The study is judged to be at high risk of bias in at least one domain for this result, or the study is judged to have some concerns for multiple domains in a way that substantially lowers confidence in the result.

**Supplementary Figure S1** Graphical representation of ROB assessment for included human studies using the Cochrane Risk of Bias 2 tool

|  | Hameroff 2013 | Bardran 2020 | Strohman 2024 | Riis 2024 | In 2024 | Legon 2024 |
| --- | --- | --- | --- | --- | --- | --- |
| Randomization process |  |  |  |  |  |  |
| Period and carryover effects |  |  |  |  |  |  |
| Deviations from the intended intervention |  |  |  |  |  |  |
| Missing outcome data |  |  |  |  |  |  |
| Measurement of the outcome |  |  |  |  |  |  |
| Selection of the reported result |  |  |  |  |  |  |
| Overall |  |  |  |  |  |  |

**Supplementary Figure S2** Percentage distribution of ROB assessment for included human studies using the Cochrane Risk of Bias 2 tool

**
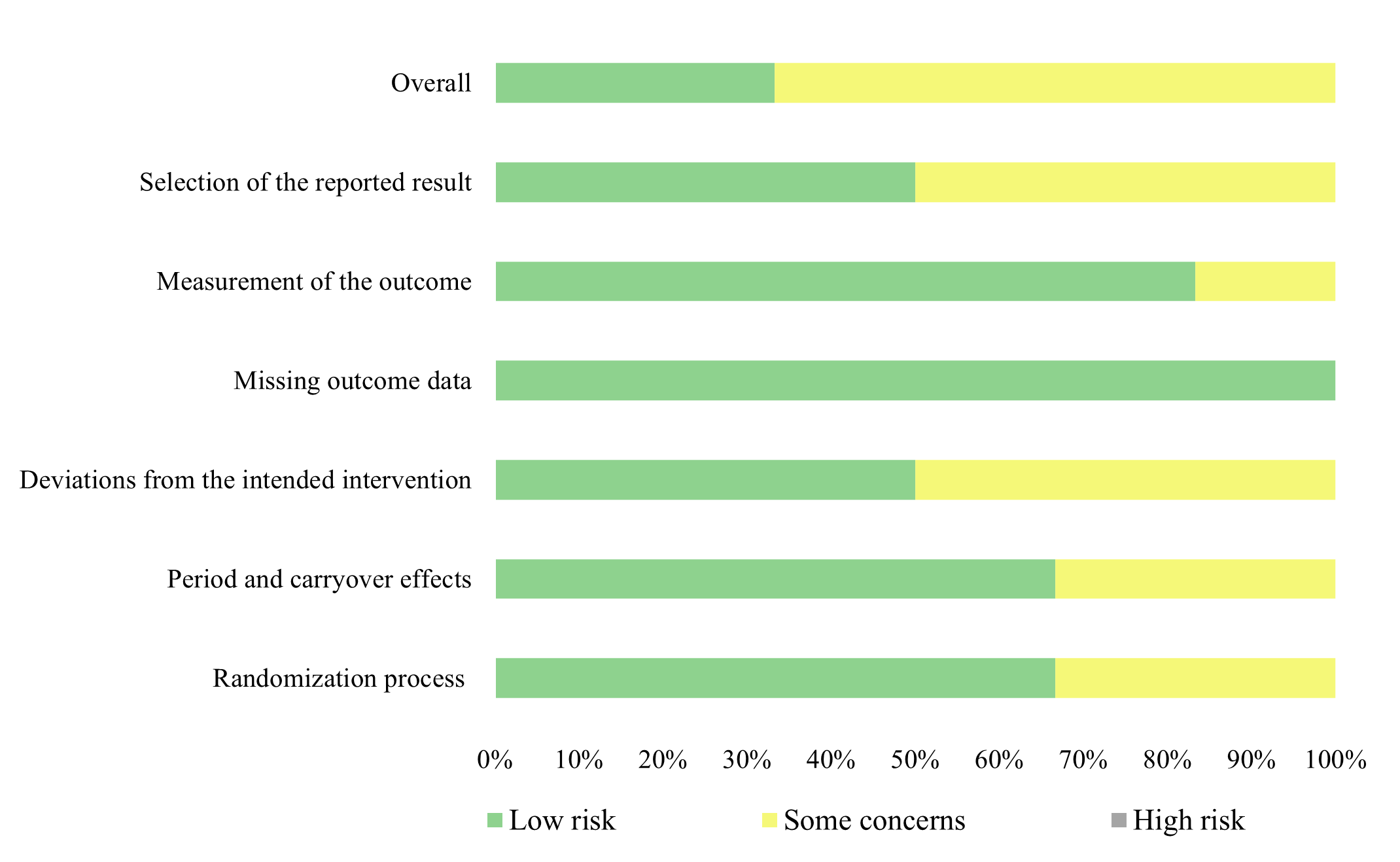
**

**Supplementary Table S2.** Full breakdown of ROB assessment for the included animal studies using the Systematic Review Center for Laboratory Animal Experimentation Risk of Bias tool

| **Author, Year** | **Selection bias** | | | **Performance bias** | | **Detection bias** | | **Attrition bias** | **Reporting bias** | **Others** |
| --- | --- | --- | --- | --- | --- | --- | --- | --- | --- | --- |
|  | **1** | **2** | **3** | **4** | **5** | **6** | **7** | **8** | **9** | **10** |
| **Feng 2021** | Unclear | Unclear | Unclear | Yes | Unclear | Unclear | Unclear | Unclear | Yes | Yes |
| **Wang 2022** | Unclear | Unclear | Unclear | Yes | Unclear | Unclear | Unclear | Unclear | Yes | Yes |
| **Zhang 2022** | Unclear | Unclear | Unclear | Unclear | Unclear | Unclear | Unclear | Unclear | Yes | Yes |
| **Mishra 2023** | Unclear | Yes | Unclear | Unclear | Unclear | Yes | Unclear | Yes | Yes | Yes |
| **Kim 2024** | Unclear | Yes | Unclear | Unclear | Unclear | Unclear | Yes | Yes | Yes | Yes |
| **Mishra 2025** | Unclear | Yes | Unclear | Unclear | Unclear | Unclear | Unclear | Yes | Yes | Yes |
| **Kim 2025** | Unclear | Yes | Unclear | Unclear | Unclear | Unclear | Unclear | Unclear | Yes | Yes |

**Criteria for each item:** Item 1: sequence generation, item 2: baseline characteristics, item 3: Allocation concealment (selection bias); item 4: random housing, item 5: blinding (performance bias); item 6: random outcome assessment, item 7: blinding (detection bias); item 8: incomplete outcome data (attrition bias); item 9: selective outcome reporting (reporting bias). item 10: other sources of bias (other bias).

**Judgement:**“Yes” indicates a low risk of bias; “No” indicates a high risk of bias; “Unclear” indicates an unclear risk of bias

**Supplementary Figure S3.** Graphical representation of ROB assessment for included animal studies using the Systematic Review Center for Laboratory Animal Experimentation Risk of Bias tool

|  | Feng 2021 | Wang 2022 | Zhang 2022 | Mishra 2023 | Kim 2024 | Mishra 2025 | Kim 2025 |
| --- | --- | --- | --- | --- | --- | --- | --- |
| Sequence generation (selection bias) |  |  |  |  |  |  |  |
| Baseline characteristics (selection bias) |  |  |  |  |  |  |  |
| Allocation concealment (selection bias) |  |  |  |  |  |  |  |
| Random housing (performance bias) |  |  |  |  |  |  |  |
| Blinding of interventions (performance bias) |  |  |  |  |  |  |  |
| Random outcome assessment (detection bias) |  |  |  |  |  |  |  |
| Blinding of outcomes (detection bias) |  |  |  |  |  |  |  |
| Incomplete outcome data (attribution bias) |  |  |  |  |  |  |  |
| Selective outcome reporting (reporting bias) |  |  |  |  |  |  |  |
| Other bias |  |  |  |  |  |  |  |

**Supplementary Figure S4.** Percentage distribution of ROB assessment for included animal studies using the Systematic Review Center for Laboratory Animal Experimentation Risk of Bias tool


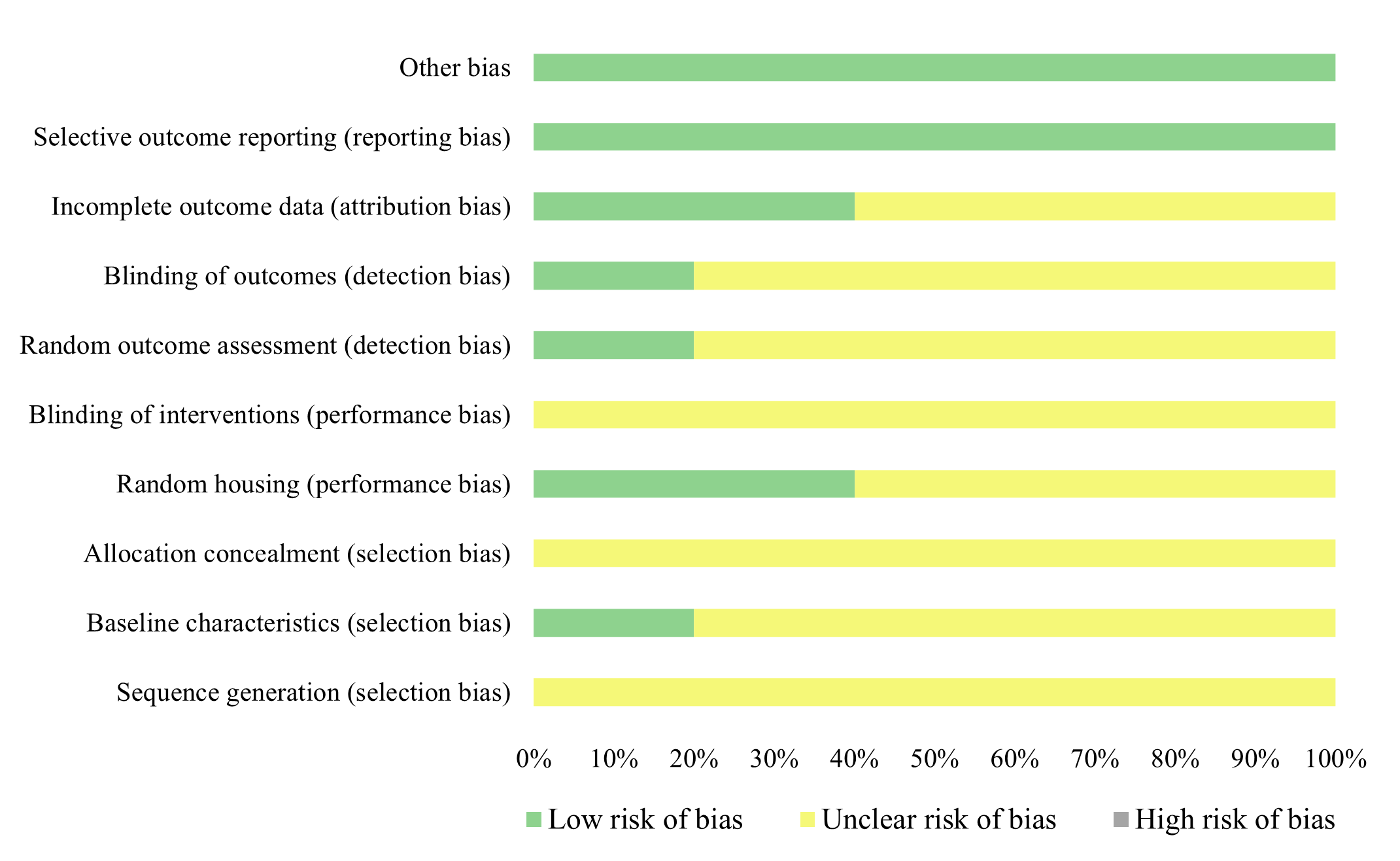

Supplement: Supplementary file 3 — Supplementary Material 3: Table S1 Full breakdown of ROB assessment for the included human studies using the Cochrane Risk of Bias 2 tool. Supplementary Table S2 Full breakdown of ROB assessment for the included animal studies using the Systematic Review Center for Laboratory Animal Experimentation Risk of Bias tool. Figure S1 Graphical representation of ROB assessment for included human studies using the Cochrane Risk of Bias 2 tool. Supplementary Figure S2 Percentage distribution of ROB assessment for included human studies using the Cochrane Risk of Bias 2 tool. Supplementary Figure S3 Graphical representation of ROB assessment for included animal studies using the Systematic Review Center for Laboratory Animal Experimentation Risk of Bias tool. Supplementary Figure S4 Percentage distribution of ROB assessment for included animal studies using the Systematic Review Center for Laboratory Animal Experimentation Risk of Bias tool. [file 10194_2025_2096_MOESM3_ESM.docx]
